# Multifunctional Flexible Humidity Sensor Systems Towards Noncontact Wearable Electronics

**DOI:** 10.1007/s40820-022-00895-5

**Published:** 2022-07-22

**Authors:** Yuyao Lu, Geng Yang, Yajing Shen, Huayong Yang, Kaichen Xu

**Affiliations:** 1 State Key Laboratory of Fluid Power and Mechatronic Systems, School of Mechanical Engineering, Zhejiang University, Hangzhou, 310027 People’s Republic of China; 2 Department of Biomedical Engineering, City University of Hong Kong, Hong Kong, People’s Republic of China

**Keywords:** Flexible electronics, Flexible humidity sensors, Noncontact detection, Healthcare monitoring, Human–machine interactions, COVID-19 epidemic

## Abstract

In the past decade, the global industry and research attentions on intelligent skin-like electronics have boosted their applications in diverse fields including human healthcare, Internet of Things, human–machine interfaces, artificial intelligence and soft robotics. Among them, flexible humidity sensors play a vital role in noncontact measurements relying on the unique property of rapid response to humidity change. This work presents an overview of recent advances in flexible humidity sensors using various active functional materials for contactless monitoring. Four categories of humidity sensors are highlighted based on resistive, capacitive, impedance-type and voltage-type working mechanisms. Furthermore, typical strategies including chemical doping, structural design and Joule heating are introduced to enhance the performance of humidity sensors. Drawing on the noncontact perception capability, human/plant healthcare management, human–machine interactions as well as integrated humidity sensor-based feedback systems are presented. The burgeoning innovations in this research field will benefit human society, especially during the COVID-19 epidemic, where cross-infection should be averted and contactless sensation is highly desired.

## Highlights

- This report summarizes recent advances of flexible humidity sensors and their integrated systems.
- Typical examples of noncontact detections based on flexible and wearable humidity sensors are highlighted.
- Research opportunities and challenges of pushing flexible humidity sensors towards practical contactless measurements are discussed.

## Graphical abstract

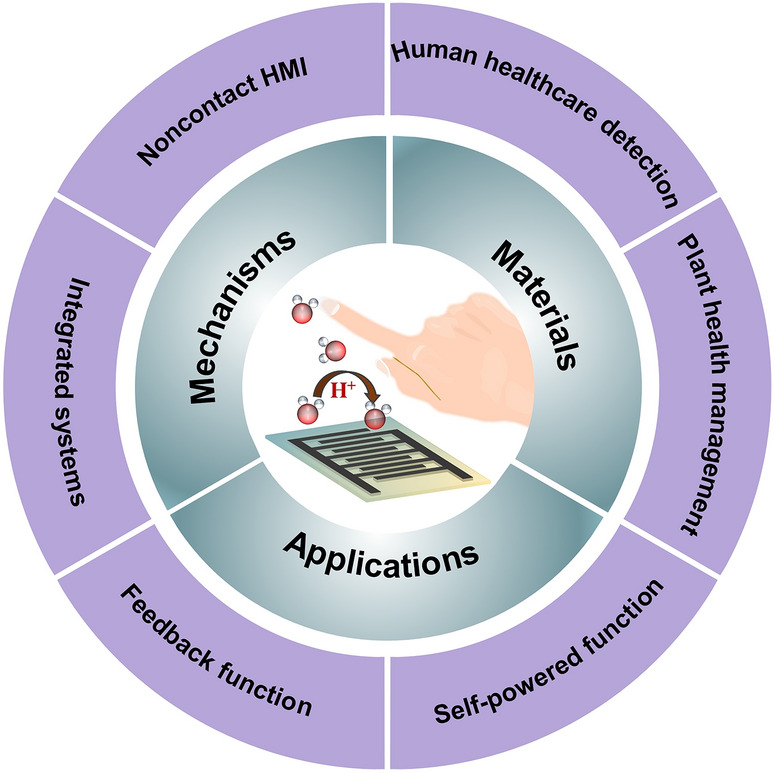

## Introduction

Skin-like electronics that imitate the multifunctional properties of human skin are emerging as rising stars for potential applications in healthcare monitoring, human–machine interfaces, implantable bioelectronics and intelligent robots [1–7]. Owing to flexible and soft nature of such skin-like devices with superior mechanical compliance, they can be seamlessly adhered onto the human skin or curved surfaces to acquire abundant information of themselves or from the ambient [8–12]. In the past decade, burgeoning functional materials and advanced manufacturing techniques have accelerated the development of a variety of skin-like sensors, which are typically categorized into flexible physical, chemical and electrophysiological sensors [13–22]. These flexible and lightweight devices contribute to bridging the human, machines and environments [23–28]. In general, conventional flexible sensors rely on signals’ detection in direct contact between the device and targets of interest, such as tactile sensors, strain sensors and most chemical sensors. Nonetheless, this tends to inevitably induce highly potential risk of cross-infections, especially in the context of COVID-19 epidemic [29–34]. Therefore, it is of great significance to explore flexible sensors with noncontact sensing capabilities. In this respect, flexible humidity sensors present such contactless perceptive functionalities by virtue of moistures that surround humid surfaces like skins, fingers, sweat and respiration etc. [35–37].

Based on a proton-hopping mechanism, a variety of active materials have been investigated for humidity sensors. These functional materials are typically endowed with superior affinity to water molecules and highly exposed surface areas so as to achieve high-performance flexible humidity sensors with high sensitivity, fast response, low hysteresis as well as superior stability [38–41]. However, a flexible humidity sensor based on unitary active materials is restricted in balancing the humidity sensitivity and response/recovery speed owing to their intrinsic properties. Therefore, various strategies, such as chemical doping, structural engineering, Joule heating, have been introduced to optimize the humidity sensing performances [42–44]. For instance, to judiciously engineer the active materials or interdigital electrodes into porous structures, it is an efficient way to absorb and desorb water vapors, thereby achieving highly stable humidity sensors [44, 45]. By doping metals, ions or nitrogen, the band structures and electrical characteristics of active humidity sensing materials can be flexibly tuned [46–48].

By means of high-performance flexible humidity sensors, they have been applied in noncontact detections owing to their capability to perceive moisture variations [37, 49, 50]. A significant application is to integrate the thin film-based humidity sensor with surgical masks for respiration monitoring [51]. Furthermore, as our skins and fingers are typically surrounded by a large quantity of moistures, physiological and psychological status can be dynamically tracked in a contactless way [52]. For example, the humidity sensors attached on the forehead, hand, chest and neck can be applied to test whether the person lies or not [53]. To prevent cross-infections, noncontact switches have been widely investigated for next-generation human–machine interfaces [35–37, 54]. Interestingly, like human beings, who regulate the body temperature by sweating, the plant can remove the heat by transpiration, which also contributes to photosynthesis and gains chemical energy. Such transpiration processes that can be accessed by the humidity sensors rely on the opening and closing of stomata to exchange water molecules with the ambient [55–57]. In addition, to satisfy the increasing demand of internet-of-things applications, research trends focus on developing integrated flexible sensor systems, which merge the multimodal sensors, flexible printed circuits as well as smart displays on the unitary system for point-of-care detections [58, 59].

**Fig. 1:**
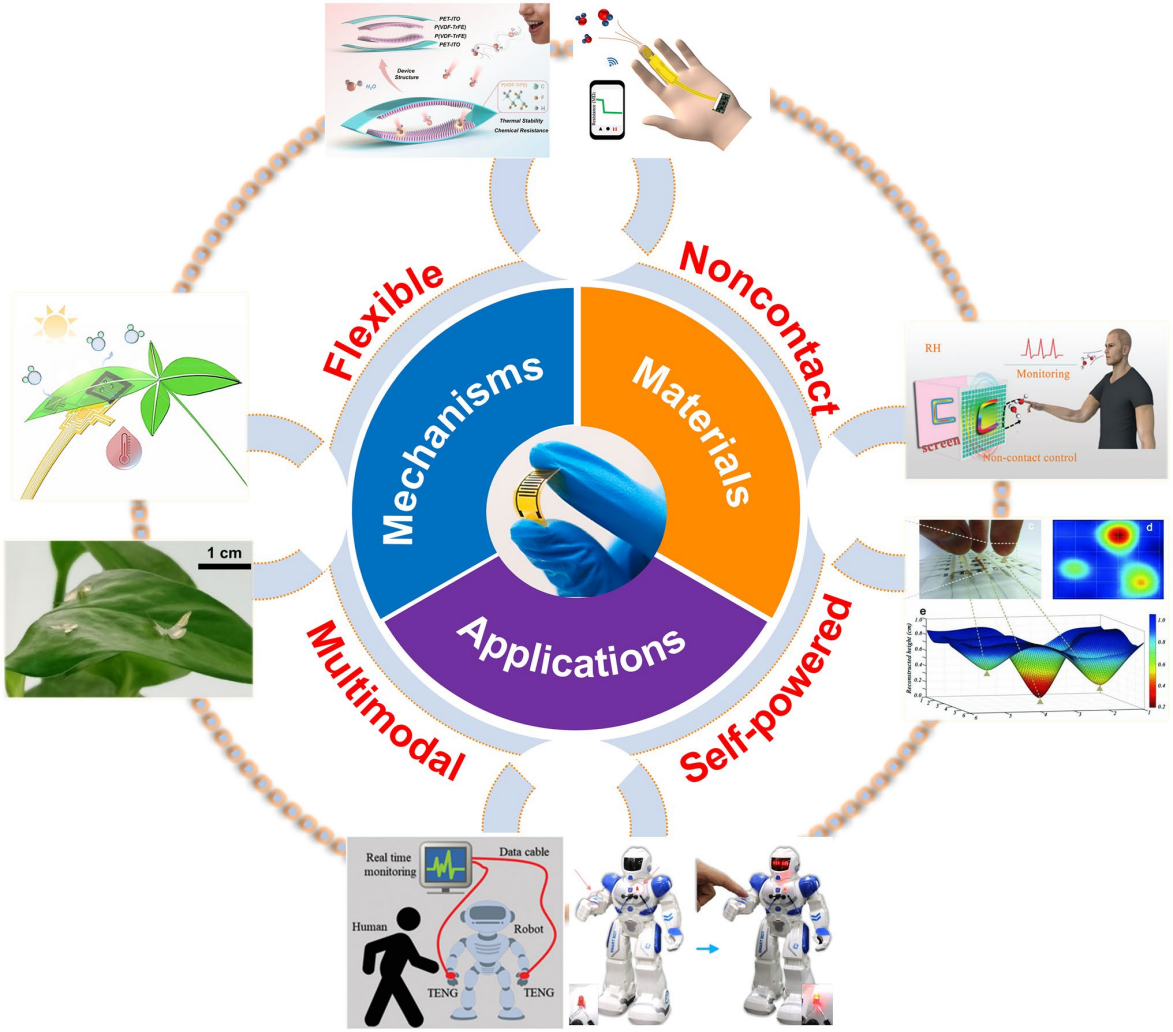
An overview of the multifunctional humidity sensors. Flexible capacitive humidity sensing system based on arc-shaped hollow structure [74]. Copyright 2021 Elsevier. Wireless finger moisture application [46]. Copyright 2021 Royal Society of Chemistry. Schematic diagram of a flexible humidity sensing system, including noncontact control and humidity monitoring [50]. Copyright 2012 Wiley. Flexible humidity sensor arrays for skin moisture mapping [100]. Copyright 2019 Wiley. The IPMEC attached on a robot as artificial skin for the detection of an approaching finger [169]. Copyright 2017 Elsevier. Schematic application of obstacle sensor in the robot for avoiding human objects [35] Copyright 2021 Wiley. Butterfly shaped sensory platform on a leaf [184]. Copyright 2018 Nature. Multimodal plant healthcare system [44]. Copyright 2020 ACS Publications

In this report, we present a timely overview of multifunctional skin-like humidity sensors from the basic materials and working principles to noncontact applications (Fig. 1). First, fundamental and working principles of flexible humidity sensors are described. Then, various categories of humidity sensors based on functional inorganic nanomaterials and polymers are highlighted. To further improve the performance of humidity sensors, several typical strategies are then introduced including chemical doping, structural design and Joule heating. Drawing on the developed humidity sensors, noncontact detections are performed, such as human/plant health monitoring, contactless human–machine interactions and feedback humidity sensor-based systems. Finally, several future trends of flexible humidity sensors are provided.

## Mechanisms of Humidity Sensors

### Fundamental Mechanism of Humidity Sensors

In principle, the basic mechanism for the majority of flexible humidity sensors can be explained by a Grotthuss chain reaction, which is usually simplified as a proton-hopping process: H_2_O + H_3_O^+^  = H_3_O^+^  + H_2_O [60–64]. It typically refers to a dynamic charge transfer process among active materials at a certain relative humidity (RH). As the signal intensity of a humidity sensor reflects the number of water molecules on the surface of sensing materials, the hydrophilic property of active materials plays a vital role regarding the sensing performance. Generally, the absorption process of water molecules could be categorized into two steps. Firstly, the first layer of water molecules is adsorbed on the surface of active materials by forming a chemical bond with the hydroxyl groups or surface defects. When RH is increased, more layers of water molecules are formed on the sensitive film via physically adsorption. Since the water molecules are easily ionized at the electrostatic field, hydronium ions are spontaneously generated and transferred among adjacent water molecules. This greatly facilitates the transfer of carriers and thus changes the output of humidity sensors.

### Different Working Principles of Flexible Humidity Sensors

Based on the aforementioned proton-hopping process, the flexible humidity sensors are generally categorized into resistive, capacitive, impedance and voltage types depending on various measurement strategies. Other humidity sensors that show responses at different frequencies like quartz crystal microbalance (QCM) [65, 66], surface acoustic wave (SAW) and Lamb wave types have been reported [67, 68], but they are usually constructed from rigid platforms. In this section, the typical working principles of flexible humidity sensors are discussed.

#### Resistive Flexible Humidity Sensor

Among a variety of humidity sensors, the resistive humidity sensors based on direct-current (DC) mode are extensively investigated due to their relatively simple and steady measurement strategy. The variation of its resistance is induced by the adsorption and desorption of water molecules on the device surface. It has been reported that the maximum resistance variation of humidity sensors is from several to thousands of percent [65, 69]. This is highly associated with electrical conductance and hydrophilicity to water molecules of active sensing materials. In addition, other representative parameters like detection range, linearity and response time are also significant.

**Fig. 2:**
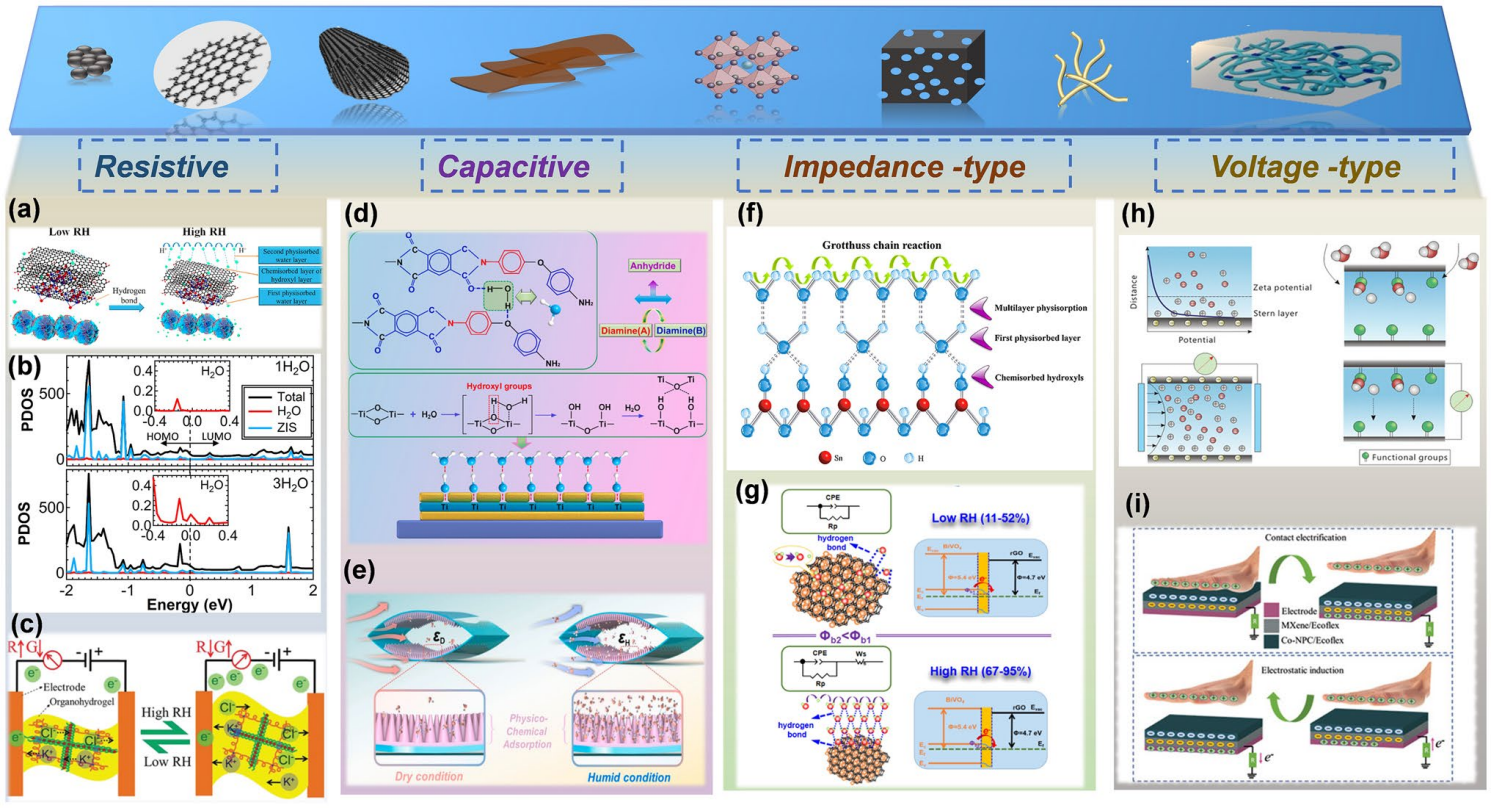
The working principle of flexible humidity sensor at low and high RH. (**a**) The adsorption of water molecules on the SnS_2_/RGO composite film at low and high RH [70]. Copyright 2019 Elsevier. (**b**) 1H_2_O and 3H_2_O at a bias of 3.3 V [44]. Copyright 2020 ACS Publications. (**c**) Schematic illustrating the humidity sensing principle of the organohydrogel [71]. Copyright 2019 Royal Society of Chemistry. (**d**) Illustration of the humidity sensing process of the water molecule adsorption [73]. Copyright 2021 Elsevier. (**e)** Schematic of the proposed flexible capacitive humidity sensing system based on arc-shaped hollow structure for monitoring human physiological signals [74]. Copyright 2021 Elsevier. (**f)** Schematic of adsorption of water layers on the SnO_2_ surface [79]. Copyright 2016 Elsevier. (**g**) Schematic diagram of the sensing mechanism [80]. Copyright 2021 ACS Publications. (**h)** Schematic mechanism for electrical generation based on streaming current and ion gradient diffusion [85]. Copyright 2020 Wiley. (**i**) Schematic of the working principle of double-layer non-contact mode TENG [35]. Copyright 2021 Wiley

Depending on charge carriers, resistive sensing materials could be divided into electronic and ionic conductive types. Electronic conductive materials are endowed with strong capability of producing electron and hole pairs. The migration of free electrons at an applied field subsequently forms a steady current. During the proton-hopping process, the hydroniums are the dominant carriers associated with the electronic conductivity on the basis of Grotthuss mechanism (Fig. 2a, b). To further figure out the physical mechanism of resistive semiconductor-based humidity sensors, first principle calculations were also carried out [44, 70]. For example, the basic mechanism of a ZnIn_2_S_4_ (ZIS) humidity sensor was uncovered by density functional theory (DFT) calculation [44]. This simulation reveals that the increased water molecules reduce the highest occupied molecular orbital (HOMO) and the lowest unoccupied molecular orbital (LUMO) gap of ZnIn_2_S_4_ nanosheets, indicating that the semiconductor property of ZIS is changed. Due to the larger tunneling effect, the higher current is generated if the device adsorbs more water molecules. Moreover, another group has calculated that 0.02 e could be transferred from one water molecule to the MoO_3_ (001) plane based on Bader charge analysis [50].

For ionic conductive materials, the conductivity depends on the liquid electrolytes distributed in polymers. Liquid electrolytes are a type of conductive compounds that could produce free ions in an aqueous solution or at a molten state. Unlike electronic conductive materials, whose charge carriers are free electrons, the current of ionic conductive material is induced by ionized cations and anions. For instance, a double network organohydrogel was designed for a high-performance humidity sensor with fast response/recovery time (0.27/0.3 s) and an over 543-fold conductance change when increasing RH from 4 to 90% [71]. The humidity response is primarily dominated by the mobility and concentrations of charge carriers (K^+^and Cl^−^ ions) (Fig. 2c). The number of charge carriers (K^+^, Cl^−^) reduces with the decreased RH due to the relatively low water content in the organohydrogel. Meanwhile, the mobility of ions is hindered due to condensed polymer chains. On the contrary, this hindering effect tends to be relieved at high RH due to the swelling of organohydrogel and the enhanced solubility of KCl, which couldgenerate more charge carriers. Both effects give rise to the high conductance.

#### Capacitive Flexible Humidity Sensor

The capacitive humidity sensor is another typical category, which shares the same basic mechanism (Grotthuss mechanism) with resistive humidity sensors (Fig. 2d). The difference is that the capacitive flexible humidity sensors usually respond to water vapors by altering the capacitance. According to device structures, general capacitive humidity sensors are mainly categorized into two types (i.e., interdigitated electrode type (IDE) and metal–insulator-metal type (MIM)) [72–74]. Both have a sandwich-like structure, which consists of two electrodes and an intermediate sensing layer in the planar for IDE and vertical orientation for MIM. Theoretically, the capacitance change is induced by the variation of dielectric constant (*ε*) of humidity sensor when it is exposed to the water vapors [74]. The capacitance is correlated with the following parameters: electrode area (*s*), thickness of sensing materials (*d*) and the dielectric permittivity (*ε*_*e*_). Since the electrostatic force constant $k$, electrode area *s* and dielectric thickness *d* are fixed, the capacitance *C* of humidity sensor is proportional to *ε*_*e*_.

1 $$C=\frac{{\varepsilon }_{e}S}{4\pi kd}$$

In addition, the humidity response of a capacitive humidity sensor can be calculated by a formula:

2 $$\mathrm{Sc}=\frac{{C}_{\mathrm{max}}-{C}_{0}}{{\mathrm{RH}}_{\mathrm{max}}-{\mathrm{RH}}_{0}}\times 100\%$$

where the Cmax refers to the capacitance at the highest RH, C0 is the capacitance at the initial RH.

As the humidity performance is strongly correlated with the dielectric constant (ε) of sensing material, the design of such dielectric layers is actually of high significance. Nevertheless, most of the IDE or MIM structure-based humidity sensors have tightly connected dielectric layers, hindering their sufficient contact with water molecules and thus relatively small capacitance variations. To enhance the sensitivity, Niu et al. demonstrated an opened arc-shaped hollow structure composed of nanocone arrays, which provides large surface areas for water vapor adsorption and relatively large space for water desorption (Fig. 2e) [74]. A comparatively rapid response/recovery time (3.693/3.430 s) is achieved. Besides the humidity effect on the output of capacitance, the capacitance value is also highly associated with the detection frequency [63, 75]. The value typically decreases with the exponentially increasing frequency.

#### Impedance-type Flexible Humidity Sensor

Impedance-type flexible humidity sensors have also been widely investigated. The sensor impedance output is highly related to the RH and frequency. Principally, the impedance gradually decreases with the increase of RH and operation frequency. The impedance curve tends to become flat at the larger frequencies, indicating that the impedance is independent of RH [76–78]. This is due to that the polarization of water molecules is hard to catch up with the rapid variation of electrical fields at the higher frequency. Generally, the humidity sensitivity presents better performance at the relatively low frequency. However, considering other properties, such as the output linearity, it is vital to investigate the RH depending on frequency. For instance, Duhan et al. demonstrated an impedance-type humidity sensor composed of Ag-doped SnO_2_ nanostructures (Fig. 2f) [79]. The impedance value presents the most linear characteristic at the frequency of 100 Hz. At this operation frequency, the sensor shows a superior reliability with a hysteresis error at 1.1%, indicating the superimposed process of adsorption and desorption. Meanwhile, He et al. reported a rGO-BiVO_4_ heterojunction-based humidity sensor (Fig. 2g). Due to the narrow bandgap of nanocomposites, the working voltage is as low as 5 mV, which is enough for the electron transfer. The impedance-frequency testing as a function of RH shows that the frequency from 1 Hz to 1 kHz has almost no effect on the sensor output. However, the impedance tends to dramatically decrease when the frequency is over 100 kHz [80]. Overall, to apply the impedance-type humidity sensors for practical monitoring, it is of high importance to find the optimal operation frequency.

#### Voltage-type Flexible Humidity Sensor

Besides the traditional flexible humidity sensors requiring continuous power supplies, battery-free and self-powered sensor systems have attracted wide research attentions [36, 81–84]. It has been widely reported that the electricity can be generated when water molecules interact with active materials [85–87]. Typically, there are two categories that account for such electricity generations, including streaming current and ion gradient diffusion (Fig. 2h). The streaming current is attributed to the movement of water molecules along a channel in a certain orientation in response to a pressure gradient or driven by a diffusive flow. As the water molecules contain cations or anions, they can be transported along with the water molecules and thereby generate electric current. The other category for power generation involves the ion concentration gradient owing to asymmetric distribution of ions on functional hydrophilic surfaces, leading to diffusion current. The output can be optimized by changing the spatial variations of moistures or engineering surface structures to induce inhomogeneous distribution of functional groups. For more fundamental physical explanations, interested readers may refer to the excellent reviewer by Shen et al. [85]. Although the moisture-enabled output power is only sufficient to drive devices with low power consumptions, it is available to achieve flexible self-powered sensors without additional power supplies, since the voltage or current output is highly associated with the RH variation surrounding devices (Fig. 2i) [35]. They have been applied to monitor human breath, moisture levels on the human skin, rain etc. In short, such voltage-type flexible humidity sensors afford a promising route to construct power-free sensing systems for a sustainable future.

## Various Functional Materials for Flexible Humidity Sensors

Generally, a humidity sensor with outstanding performance mainly relies on the property of active materials, which are considered as the core of humidity sensors [88, 89]. Recently, functional inorganic nanomaterials, such as carbon, metal sulfides and metal oxides, stand out from diverse active materials for flexible humidity sensors due to their properties of highly exposed surface area and superior affinity to water molecules. Furthermore, functional polymer-based flexible humidity sensors gain extensive research attentions because of their biocompatible and biodegradable characteristics. In this section, recent advances of flexible humidity sensors based on inorganic nanomaterials and polymers are discussed.

### Inorganic Nanomaterials

#### Carbon Materials

**Fig. 3:**
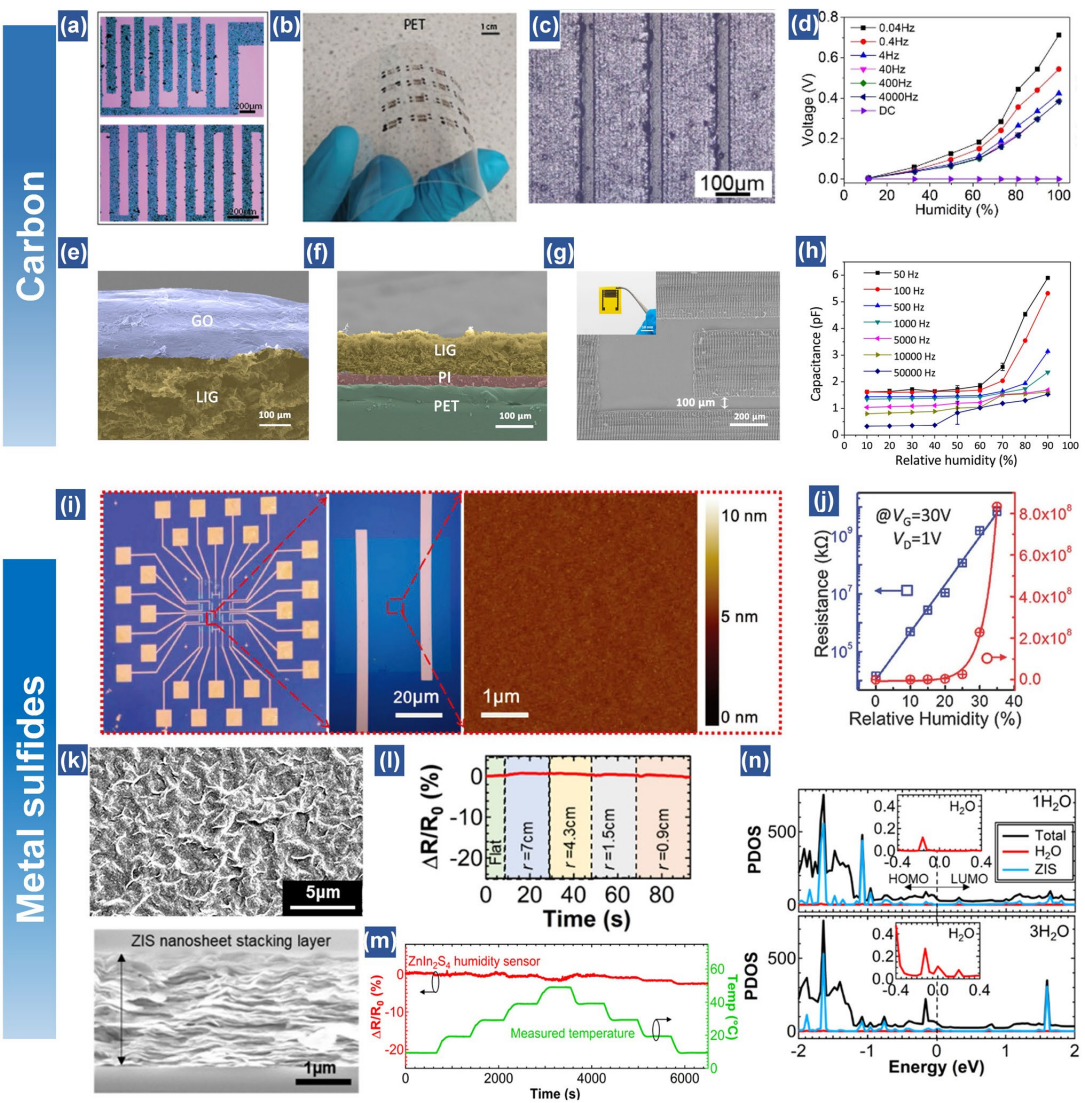
Carbon materials-based and Metal sulfides-based flexible humidity sensors. (**a**) Optical microscope image of the resulting graphene pattern. (**b**) All-graphene devices built on PET substrate with carbon paste contacts (all-carbon devices) [96]. Copyright 2019 Wiley. (**c**) Interdigitated pattern of rGO/GO/rGO prepared on a flexible PET film and corresponding optical microscope image. (**d**) The change of the sensing peak voltages toward RH at different frequencies [97]. Copyright 2018 ACS Publications. (**e**) Cross-sectional SEM image of the GO-based sensor. (**f**) Cross-sectional SEM image of the PI-based sensor. (**g**) SEM images of the PI-based sensors showing finger spacing of 100 μm. (**h**) Relationship between capacitance and RH at different frequencies of the PI-based humidity sensors using LIG-IDEs with finger spacing of 100 μm [98]. Copyright 2020 Elsevier. (**i**) A typical 3D AFM image showing the edge of the WS_2_ film on the SiO_2_/Si substrate. Black line: A typical height profile showing the thickness of the WS_2_ film. (**j**) Response of the sensor in different relative humidities. Inset: The magnified curve of the low RH region from 20 to 45% [104]. Copyright 2017 Royal Society of Chemisty. (**k**) SEM images of surface and cross-sectional views of the ZnIn_2_S_4_ film. (**l**) Bending test of the flexible ZIS humidity sensor under RH 30%. (**m**) Temperature dependence test of the ZIS humidity sensor under temperature change from 10 to 50 ℃ at RH 30%. The temperature was measured by a commercial temperature sensor in the oven. (**n**) Structural configuration of the simulation model. Projected density of states (PDOS) with 1H_2_O and 3H_2_O at a bias of 3.3 V [44]. Copyright 2020 ACS Publications

Among a variety of inorganic nanomaterials, graphene and its derivatives (*i.e.,* graphene oxide (GO) and reduced graphene oxide (rGO)) have been extensively investigated as humidity or gas sensors owing to their large surface area, low toxicity, mechanical compliance and good chemical stability [36, 47, 60, 90, 91]. The abundant active sites, such as defects, vacancies and hydrophilic groups on their surfaces are able to capture water molecules, contributing to desired humidity perception. Typically, GO is applied in capacitive humidity sensors due to it electrical insulator properties, while rGO is employed in resistive humidity sensors owing to its resistance sensitive to the humidity change. Since an early report by Borini et al. [92], GO and rGO-based humidity sensors or sensor arrays have been widely demonstrated for human respiration monitoring, and non-contact sensation [90, 93–96]. For instance, GO can be spray-coated on a commercial silk fabric for a wearable respiration mask [95]. The device exhibits almost no performance degradation after 2500 cycles’ bending or twisting. Furthermore, Craciun et al. demonstrated a wafer-scale all-graphene-based humidity sensor on silicon and poly(ethylene terephthalate) (PET) surfaces based on the techniques of pattern generation and material deposition (Fig. 3a, b) [96]. The processes are compatible with complementary metal oxide semiconductor (CMOS)-based methods, presenting promising industry applications.

Besides the traditional mask-based approach to realize the humidity sensor array, laser direct writing (LDW), as a mask-free technique is also a promising route to pattern the nanomaterials. Cai et al. demonstrated a rGO/GO/rGO configuration realized by LDW for humidity sensing (Fig. 3c, d) [97]. Primarily based on photothermal effect, GO can be selectively converted to rGO with high conductivity and porosity. The device relies on an AC sensing mode, which measures output voltage rather than traditional impedance or capacitance. This results in humidity detection range from 6.3 to 100% RH. Another group demonstrated a laser fabrication method to create the interdigital laser-induced graphene (LIG) electrodes (Fig. 3e–g) [98]. The advantage is that the LIG is directly produced via laser carbonization on a polyimide film without loading precursors. After that, the GO solution was drop-casted on the electrodes as the active material sensitive to moisture (Fig. 3e–g). The output of sensor presents a rising trend as RH increases from 10 to 90%, while the entire curve shows a downward trend as the frequency increases (Fig. 3h). This matches well with the working mechanism of capacitive humidity sensors. To further improve the sensing performance, to construct heterogenous nanocomposite is an excellent strategy. For example, the proposed alternative poly(dopamine) (PDA)/graphene multilayers show high sensitivity, ultrafast response and wide humidity detection range (0–97% RH) due to the high specific surface area [53]. The interlayer distance of polymer can be flexibly tuned by graphene sheets from 0.7 to 1.4 nm. An over four orders of sensitivity enhancement is achieved. More importantly, the sensor shows little hysteresis for humidity detection with a superfast response (20 ms) as well as recovery time (17 ms). Such outstanding performance is attributed to the thin polymer layer decorated with PDA chains and high electrical conductivity of graphene, leading to the rapid adsorption of water molecules and immediate release of water molecules at the low humidity.

#### Metal Sulfides

Owing to the large surface area and multi-layered structures, many other nanomaterials are also widely utilized as humidity sensing media, such as sulfide-based nanomaterials like MoS_2_, VS_2_, WS_2_, ZnIn_2_S_4_, CdS etc. [44, 91, 99–102]. The common advantages of these nanomaterials in humidity sensing are their rich hydrophilic surface sites and tailored band structures, which allow the occurrence of proton transition induced by the absorbed water molecules as long as the humidity level is increased. Additionally, due to the ultrathin 2D layered semiconductors, it is possible to create transparent humidity sensors. For instance, WS_2_ films (thickness: ~ 2.1 nm) can be patterned using graphene as electrodes on a thin PDMS membrane, allowing the formation of transparent and stretchable humidity sensors [103]. The device can reach a high humidity response (up to 2,357) at a RH of 90% and present 40% elastic stretch. Furthermore, Zhao et al. realized a humidity sensor array based on a single-layer MoS_2_ (Fig. 3i, j) [104]. Although the range of relative humidity perception is from 0 to 35%, the sensitivity is over 10^4^, which can be judiciously tuned by the gate voltages. In addition, the humidity sensing behavior could also be optimized via building *p*-*n* junctions between p- and n-type semiconducting materials. Based on different Fermi-levels of two semiconductors, a *p*–n junction is successfully built at the contact interface of *n*-type SnS_2_ and *p*-type RGO [60]. Impressively, the humidity sensitivity of SnS_2_/RGO based humidity sensor is nearly 12 times higher than that of pristine SnS_2_ based humidity sensor. This is because that the barrier at the contact interface of two materials becomes lower at high humidity atmosphere, which is triggered by the proton-hopping induced on the composite film surface.

Another important property of humidity sensor that is vital for practical applications is the flexible stability, which requires the sensing materials with desired mechanical compliance to ensure the stable performance during measurements, especially when the devices are attached on human skin or other curved surfaces. For example, a ZnIn_2_S_4_ nanosheets-based humidity sensor is found with well-stacked and wrinkled surface structures, which allows the device to show outstanding mechanical stability at different bending curvatures (Fig. 3k-m) [44]. In addition, this resistive-type ZnIn_2_S_4_ humidity sensor presents nearly no resistance change (< 5%) as the temperature is increased to 50 °C, denoting a low cross-coupling effect of humidity response with other signals. Moreover, as the ZnIn_2_S_4_ nanosheets are applied as the humidity sensor for the first time, first-principles calculations are applied to reveal the mechanism. It is found that the increased current intensity of humidity sensor is attributed to the large tunneling effect at relatively high humidities (Fig. 3n).

#### Metal Oxides

**Fig. 4:**
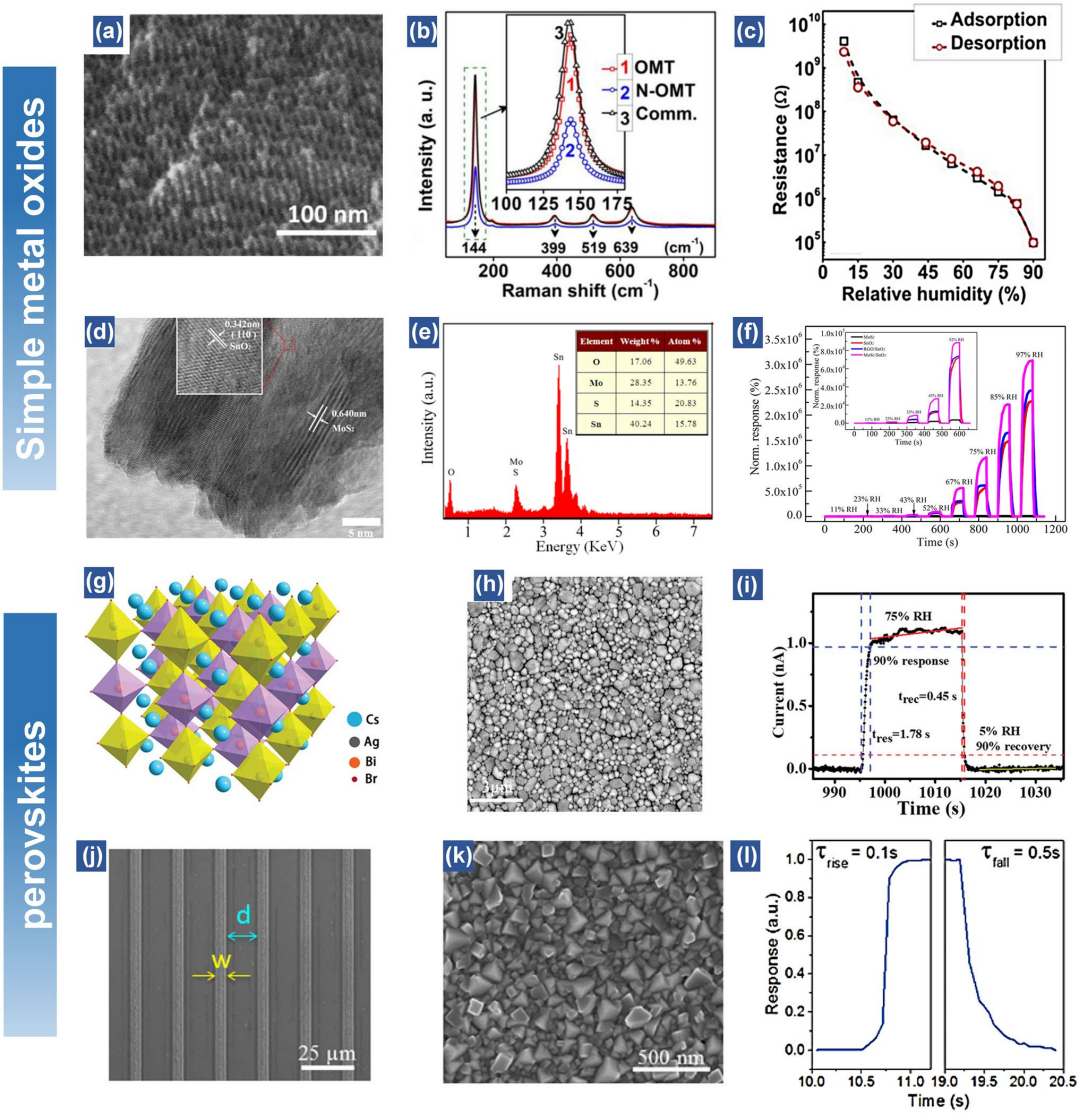
Simple metal oxides-based and perovskites-based flexible humidity sensors. (**a**) SEM images of nitrogen doped ordered mesoporous TiO_2_ (N-OMT). (**b**) Raman spectra of OMT, N-OMT and Commercial TiO_2_. (**c**) Hysteresis of sensor based on N-OMT [48]. Copyright 2018 Elsevier. (**d**) TEM images of MoS_2_/SnO_2_ hybrid. (**e**) EDS spectrum and elemental composition of the MoS_2_/SnO_2_ hybrid. (**f**) Comparative results of a MoS_2_/SnO_2_ hybrid film sensor in sensing response with pure MoS_2_, pure SnO_2_, and RGO/SnO_2_ hybrid film. Inset: the comparative normalized response of the four sensors exposed to 11 − 52% RH [78]. Copyright 2016 ACS Publications. (**g**) Schematic structure of Cs_2_BiAgBr_6_ crystal. (**h**) Top-view SEM image of Cs_2_BiAgBr_6_ film. (**i**) One dynamic response and recovery curve under 1 V bias [41]. Copyright 2019 Wiley. (**j**) spin-coated PbI_2_ and CH_3_NH_3_PbI_3_ microstripes with widths of 5 μm. (**k**) Surface of the CH_3_NH_3_PbI_3_ microstripes. (**l**) Transient response at 95% RH for perovskite microstripes [69]. Copyright 2019 ACS Publications

Similar to metal sulfides, metal oxides-based nanomaterials also exhibit hydrophilic property for sensitive humidity sensors [105]. Owing to the relatively narrow bandgap nature, metal-oxides nanomaterials with excellent electronic properties are mainly applied for resistive or impedance-type flexible humidity sensors [80, 106]. Until now, various metal oxides including TiO_2_, ZnO, CuO, SnO_2_, MoO_3_, and HNb_3_O_8_ have been applied as the active materials in response to humidity changes [46, 50, 75, 107–109]. For example, Shen et al. synthesized MoO_3_ nanosheets as active materials for transparent humidity sensors [50]. A humidity response of over 10^4^ times when increasing RH from 0 to 100% is obtained along with a rapid response/recovery time (< 0.3/ < 0.5 s). To further improve the sensing performance, surface modification methods are excellent strategies to enhance the physicochemical properties of active materials. For instance, Li et al. reported the nitrogen-doped mesoporous TiO_2_ as the humidity sensing matrix with a superior sensitivity (resistance change > 10^5^) [48]. The high humidity response is related to the lattice oxygen (Ti–O) and hydroxy (Ti–OH) in the crystal structure when exposed to water molecules (Fig. 4a). Raman results reveal the distortion of TiO_2_ lattice after nitrogen-doping, which facilitates the substitution of oxygen in the defects (Fig. 4b). The results indicate that the nitrogen-doping and mesoporous structure are synergistically conducive to the optimization of humidity sensitivity and hysteresis (Fig. 4c). In another research, Zhang et al. demonstrated a capacitive humidity sensor based on MoS_2_-modified SnO_2_ nanocomposite. The crystallinity and oxygen-rich nature of this nanocomposite makes contributions to the capacitance change (Fig. 4d). The high composition of oxygen atoms in this hybrid material system facilitates the water adsorption process, leading to the enhanced sensing performance of the composite (1,167,620%) compared to the pristine SnO_2_ nanosheets-based humidity sensor (572,318%) when exposed to 75% RH (Fig. 4e, f) [78].

#### Perovskites

Besides the simple metal oxides, perovskites are also found with superior humidity sensing properties. In the past decades, diverse perovskite materials are reported with exceptional properties including superconductivity, ferroelectricity, high carrier mobility, decent thermal stability, etc. [110]. These properties allow the perovskite-based materials to explore more functionalities towards various application fields, such as solar cells, gas and humidity sensors, transistors, and light emitting diodes [111–114]. In terms of the perovskite for humidity sensors, due to the existence of abundant oxygen and oxygen vacancies on the surface, metal oxide perovskites including Bi_3.25_La_0.75_Ti_3_O_12_, BaTiO_3_ and NaNbO_3_ are quite sensitive to water molecules [115–117]. In comparison, the metal halide perovskites such as Cs_2_BiAgBr_6_ and CH_3_NH_3_PbI_3_ present a weaker hydrogen bond than that of metal oxide perovskites, resulting in a relatively low humidity sensitivity [41, 69]. However, this property facilitates the desorption of attached water molecules and thus shortens the recovery time. For example, Zhan et al. employed a lead-free perovskite, Cs_2_BiAgBr_6_ thin film as active humidity sensing materials [41]. The device demonstrates a much faster response (1.78 s) and recovery (0.45 s) time (Fig. 4g–i). Such performances are much better than the most reported humidity sensors. Wu et al. proposed a microstripe CH3NH3PbI_3_-based humidity sensor, presenting response and recovery time of 0.1 and 0.5 s, respectively [69]. The resistance change can be decreased by four orders when the humidity level changes from 10 to 95% (Fig. 4j–l). In short, the perovskites-based humidity sensors show superior humidity sensing performance. However, to meet the requirements of practical applications, the brittleness nature that may influence the mechanical stability under bending, together with the instability at high humidity levels remains to be resolved.

### Polymers

With the increasing demands of biocompatible and environmental-friendly flexible electronics, polymers including bio-inspired and biodegradable materials [54, 118–120], have attracted extensive research attentions to serve as building blocks for various sensors. Owing to the high transparency, super-elasticity, outstanding mechanical properties as well as biocompatibility of polymers, their economically practical applications can be found everywhere in our life [121, 122]. Basically, most of polymers are linked with various functional groups, such as –COOH, –OH, and –NO_2_ [123]. The humidity sensing property of polymers is mainly determined by the hydrophilic performance of functional groups, which could form rich hydrogen bonds with water molecules [65]. Basically, polymer can be classified into natural polymer and synthetic polymer. Natural polymers endowed with linear long chains as the basic structures, is widely distributed in animals and plants. To improve the physical and chemical properties of natural polymer, various synthetic polymers, especially conductive polymers are fabricated. Depending on the distinct charge carriers, conductive polymers can be categorized into electron-conductive polymer and ion-conductive polymer.

#### Natural Polymers

**Fig. 5:**
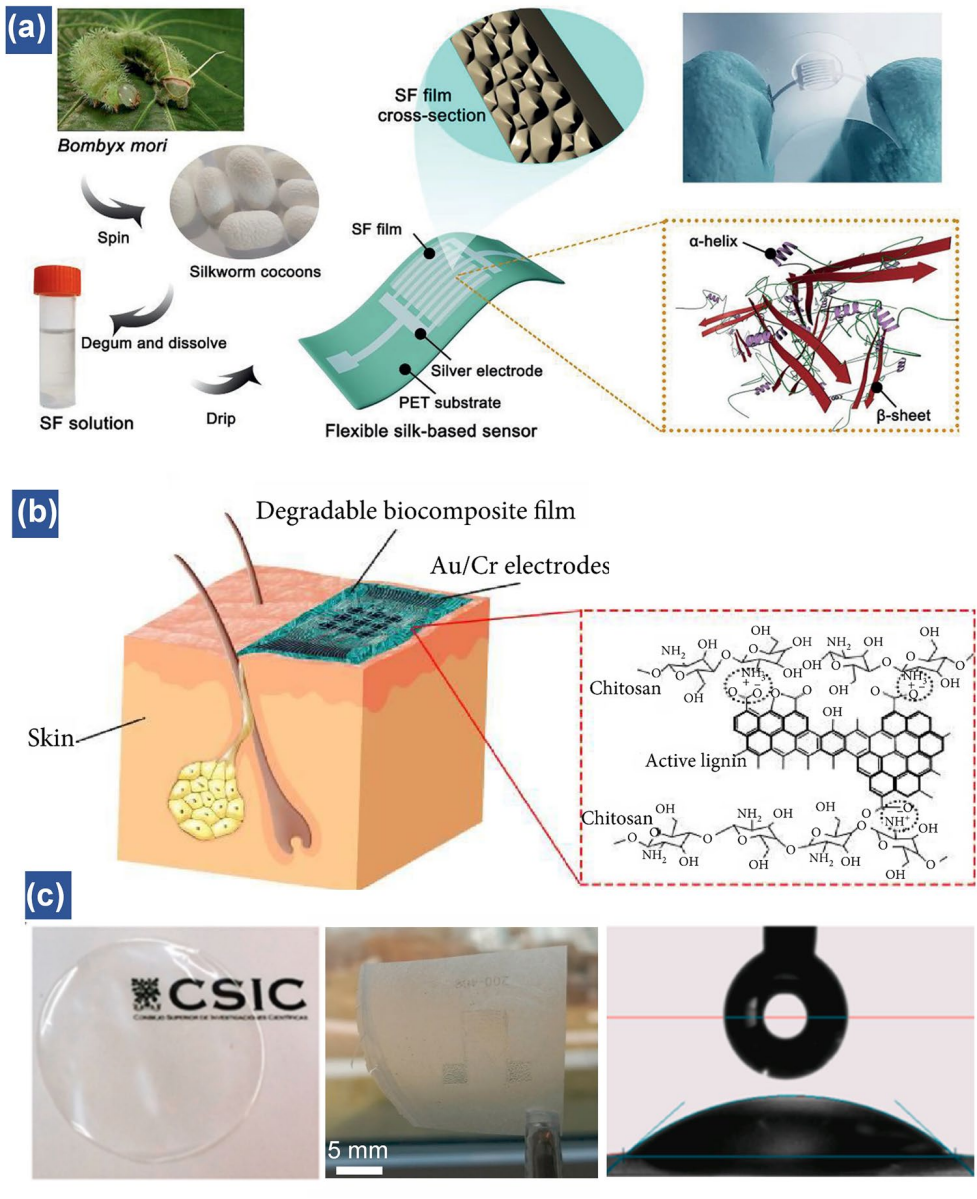
Natural polymers-based flexible humidity sensors. (**a**) Schematic illustration showing the fabrication of the SF-based humidity sensor. Insets illustrate cross-section and structure of the natural SF film used to fabricate the sensor [126]. Copyright 2020 Wiley. (**b**) Schematics of a biodegradable and biocompatible humidity sensor attached on the skin. The inset shows the chemical structure of natural functionalized polysaccharide film used to fabricate the sensor [54]. Copyright 2020 Science Partner Journal. (**c**) Optical photographs and water contact angle of CNF films [130]. Copyright 2021 Elsevier

Owing to the intrinsic hydrophilic property, a variety of natural biomaterials, such as cellulose and proteins, are found with excellent humidity performance including high humidity response, outstanding flexibility and remarkable noncontact sensation [40, 54, 124, 125]. A promising path to achieving highly responsive humidity sensors is to utilize or mimic the natural products. Inspired by the bionic structure of silkworm cocoons, the transparent silk fibroin films endowed with superior optical property and electrical conductivity were uniformly coated on the interdigital silver electrodes on a PET substrate (Fig. 5a) [126]. The optimum thickness of silk fibroin (SF) films for the humidity measurement is around 23 μm. Different from the conventional humidity sensors, the proposed silk-based humidity sensor can exhibit visual color changes at different humidity levels.

One of the unique properties of biomaterials-based humidity sensors is their biodegradability, which ensures the safety of sensors upon wearing on human body for a long time. Generally, the degradation of flexible sensors is triggered by physical or chemical stimuli such as ultraviolet light irradiation, heating and pH [54, 127–129]. Among them, the acid-driven degradation plays a significant role in decomposing natural polymers into oligomers by acid hydrolysis. The decomposed monomers can be easily broken down in the natural environment with the help of microorganisms or the organisms in human immune systems. As illustrated in Fig. 5b, the polymer-based humidity sensor attached onto the human skin is composed of active chitosan composite film [54]. In medicine, chitosan and its composites are widely utilized as wound-dressings due to their high biocompatibility and antibacterial properties. Owing to the high solubility of chitosan composite films in an acidic aqueous solution with pH value of 5.5 at room temperature, this flexible humidity sensor could be selectively decomposed to small molecules in a particular circumstance. Meanwhile, to achieve a completely degradable flexible humidity sensor, the disintegrable Fe/Mg metal system is chosen as the electrodes instead of Au/Cr due to easy-processing and rapid hydrolysis properties. Another recent work investigated a cellulose nanofibers (CNF)-based humidity sensor, which presents high transparency and hydrophilicity due to the abundant OH^−^ and COON groups distributed on the CNF films (Fig. 5c) [130]. To improve the conductivity of natural polymer, conductive nanomaterials are usually introduced to enhance the electron transfer. 2,2,6,6-tetramethylpiperidine-1-oxyl (TEMPO)-oxidized cellulose fibers in conjunction with CNTs enable the formation of humidity sensor with an outstanding linearity (*R*^2^ = 0.995) between 11 and 95% RH and commendable stability for over three months [131]. The adsorption sites for water molecules are provided by hydrophilic hydroxyl groups on cellulose fibers, contributing to the electron transfer between CNTs and water molecules. Overall, it is significant to develop the green and biocompatible flexible electronics based on natural materials and their composites, especially for wearable devices on human body [132]. *3.2.2. Synthetic Conductive Polymer.*

**Fig. 6:**
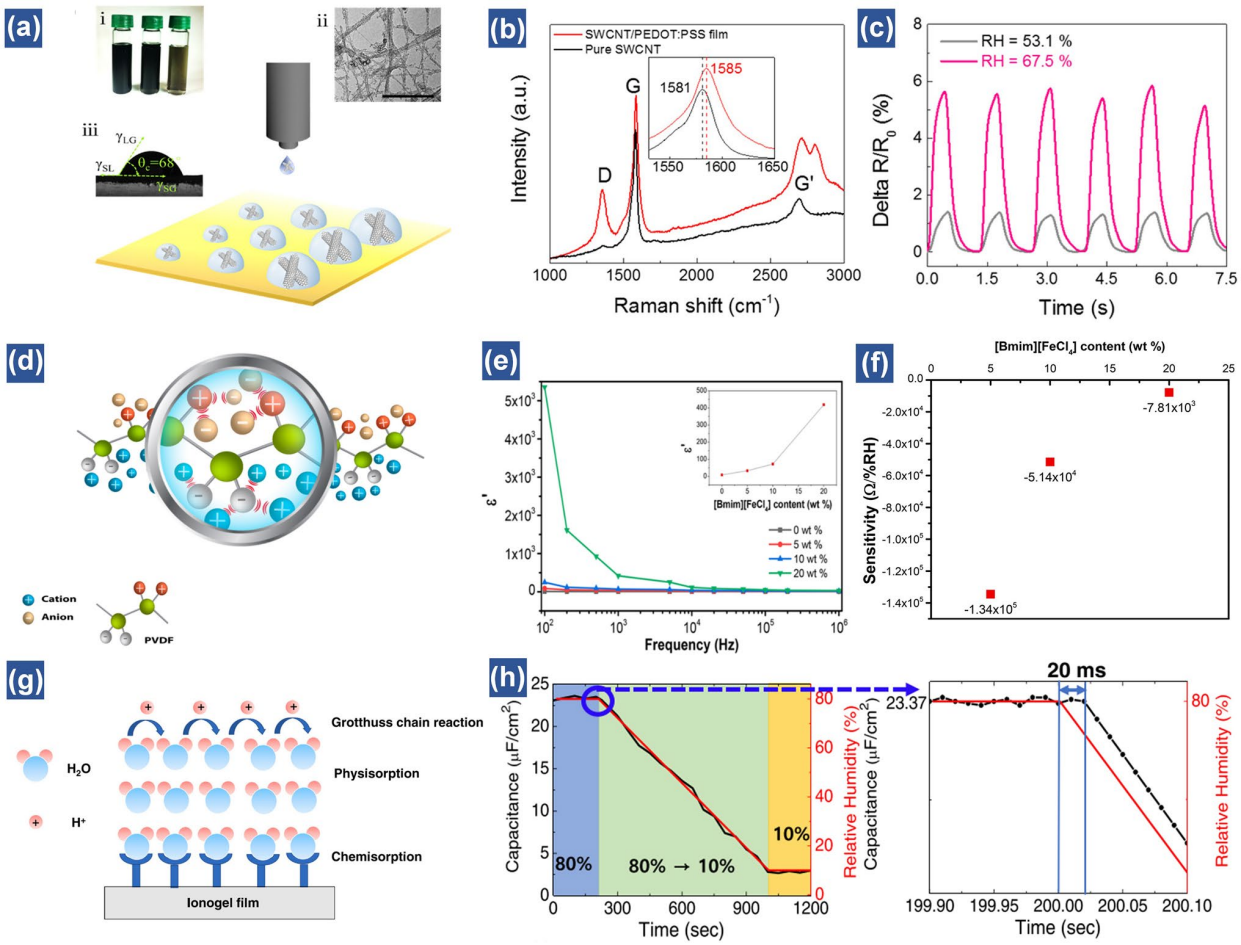
Synthetic conductive polymers-based flexible humidity sensors. (**a**) Schematic illustration of the preparation of SWCNT/PEDOT:PSS hybrid films through CRL. Insets are SWCNT/PEDOT:PSS hybrid inks with different concentrations (0.1, 0.2, and 0.3 mg mL^−1^, from right to left) (i), and their TEM image (ii), and contact angle on PET substrate (iii); scale bar is 50 nm. (**b**) Raman spectra; inset shows the shift G peak. (**c**) Short cyclic tests of humidity-resistance response with different RH value or distance [141]. Copyright 2015 ACS Publications. (**d**) Ion − dipole interactions between PVDF and [Bmim][FeCl_4_]. (**e**) PVDF/IL composites electrical response of dielectric constant (**f**) Sensor sensitivity as a function of the [Bmim][FeCl_4_] content [143]. Copyright 2019 ACS Publications. (**g**) Schematic of the humidity sensing mechanism of the PIL. (**h**) Capacitance as a function of the measuring time with respect to the increase in RH from 10 to 80% [147]. Copyright 2021 Elsevier

In recent years, the development of synthetic conductive polymers in flexible electronics has gained much attention due to the highly physicochemical stability and conductivity [133]. According to the category of charge carriers in polymer networks, synthetic conductive polymers can be divided into electron-conductive polymer and ion-conductive polymer. Intrinsic electron-conductive polymers are endowed with conjugated long chains, where the delocalized *π* electrons on the double bond can migrate to form a current. Typical intrinsic conductive polymers refer to polyaniline (PANI), polypyrrole (PPy) and polythiophene (PEDOT) etc. [66, 134, 135]. For example, the humidity sensing behavior of a PANI nanofibers-based humidity sensor shows decrease in resistance at low humidity levels (0–52%), while the resistance presents an inverse trend at high humidity levels (50–84%) [136]. The reduction of resistance at low RH is attributed to the efficient charge transfer during the water absorption process. By further increasing the RH, the swelling phenomenon takes place in the polymer chains, which restricts the charge carrier movement, therefore resulting in the reverse response. However, this bimodal humidity property is undesired. Sandhu et al. engineered the PANI into nanogranular structures, which give rise to a unimodal humidity response property with a linear characteristic from 16 to 96.2% RH [137]. This is probably ascribed to the swelling-resistant morphology of nanogranular shapes. Another strategy to improve the moisture response relies on mixing polymer with inorganic materials, such as metals, carbon materials and conductive fibers to form conductive composites [138–140]. For example, Tai et al. proposed a novel coffee-ring lithography method to fabricate the single-wall carbon nanotube (SWCNT)/poly(3,4-ethylenedioxythiophene)-polystyrene sulfonate (PEDOT: PSS) hybrid ink for applications in transparent humidity sensors. Owing to the high surface hydrophilicity of both materials, this hybrid film shows evident resistance change when RH varies from 53.1 to 67.5% (Fig. 6a–c) [141]. Such a transparent hybrid film with the capability to perceive humidity variations demonstrates the potentials in noncontact screens.

Another group of conductive polymer is ion-conductive polymer. The main difference between electron-conductive polymer and ion-conductive polymer lies in the charge carriers. The charge carriers in electron-conductive polymer are free electrons while in ion-conductive polymer are ions. Generally, polymer composites consisting of liquid electrolytes are conceived as typical ionic conductors. The common liquid electrolytes mainly include aqueous electrolytes (such as KCl, KOH, and CoCl_2_) and ionic liquids (such as 1-ethyl-3-methylimidazolium bis(trifluoromethylsulfonyl)imide ([Emim][TFSI]) and 1-butyl-3-methylimidazolium tetrachloroferrate ([Bmim][FeCl_4_]) [142–145]. As a kind of green solvent, ionic liquids show good solubility and miscibility with various organic and inorganic materials [146]. Moreover, the long alkyl chain structure allows them to be engineered with suitable properties including conductivity and hydrophilicity [145]. For instance, an ionic polymer film consists of poly(vinylidene fluoride) (PVDF) and ionic liquid [Bmim][FeCl_4_] was developed for humidity sensing applications (Fig. 6d). Upon incorporating the ionic liquid with PVDF, the frequency dependent dielectric constant goes up along with the increasing content of ionic liquid, leading to enhancement of electronic conductivity (Fig. 6e). As a result, the resistance of this composite decreases with the increasing concentration of ionic liquid from 5 to 20%, resulting in the decrease of sensitivity from 134,485 to 7,808 Ω/% RH (Fig. 6f) [143]. To meet the demand of emerging sensor applications, Ha et al. investigated a capacitive humidity sensor based on a bar-printed Poly(ionic liquid) (PIL) by embedding 1ethyl-3-methylimidazolium bis(trifluoromethylsulfonyl)imide ([EMIM] [TFSI]) in poly(methyl methacrylate) (PMMA). At a high RH, free proton hopping occurs on the composite surface, leading to the increase of capacitance (Fig. 6g). Notably, the ultrafast response and recover time (20 ms) can be achieved due to the superior hydrophobic property of ionic liquid (Fig. 6h) [147]. Overall, via optimizing the structures and hydrophilicity of polymer by different strategies, it is feasible to obtain a conductive polymer-based humidity sensor with outstanding performance in response to moisture variations. Table 1 compares various flexible humidity sensors in terms of active materials, electrodes, working mechanisms and properties.

**Table 1:** The performance comparison results of different categories of flexible humidity sensors

Sensing Material	Category	Working mechanism	Electrodes	Maximum sensitivity	Response/recover time (s)	Detection range (RH%)	Linearity	Cyclic performance (cycles)	Application	Year	References
GO	Carbon material	**Resistive**	PEDOT:PSS/Ag colloids	~ 3% (ΔR/R_0_)	50/421	12–97%		5 (12%-59%)	Breathing test	**2018**	[90]
Pt-nRGO fiber	Carbon material	**Resistive**	Au	3.53% (ΔR/R_0_)	**0.064/0.508**	6.1–66.4%	**Linear**		Breath monitoring	**2018**	[47]
MoS_2_	Metal sulfides	**Resistive**	Ti/Au	**10**^**4**^ (R/R_0_)	10/60	0–35%	**Linear**	5 (0–10%)	Finger moisture mapping	**2017**	[104]
WS_2_	Metal sulfides	**Resistive**		**469%** (∆I/I_0_)	12/13	11–97%	Nonlinear	4 (11.3%-97.3%)		**2016**	[101]
VS_2_	Metal sulfides	**Resistive**	Au	** ~ 200%** (ΔR/R_0_)	30–40/12–50	**0–100%**	Nonlinear	4 (10%-60%)	Fingertip moisture detection	**2012**	[100]
Pd/HNb_3_O_8_	Metal oxides	**Resistive**	LIG	~ 40% (ΔR/R_0_)	**0.2/3**	30–99.9%	Nonlinear	**50 (30%-90%)**	Dehydration test	**2021**	[46]
MoO_3_	Metal oxides	**Resistive**	ITO	**10**^**3**^ (R/R_0_)	**0.5/2**	**0–100%**	**Linear**	5 (0–40%)	Finger moisture mapping	**2019**	[50]
Silk fibroin	Natural polymer	**Resistive**	Ag	**750%** (ΔR/R_0_)	73.1/11.3	43–95%	Nonlinear	5 (59%-75%)	Respiration monitoring and smart noncontact sensing	**2021**	[126]
Ti_3_C_2_ -derived TiO_2_	Metal oxides	**Capacitive**	Au	** ~ 1614 pF/%RH** (> 33% RH)		**7–97%**	Nonlinear	6 (7%-33%, 7%-54%, 7%-84%)	3D mapping of the approaching fingertips	**2019**	[75]
[P(VDF-TrFE] nanocone arrays	Polymer	**Capacitive**	ITO		**3.693/3.4**	50 − 90%	**Linear**	3 (50%-90%)	Physiological signals monitoring	**2021**	[74]
GO	Carbon material	**Capacitive**	LIG	**3215.25 pF/%RH**	15.8	10–90%		6 (20%-80%)	Long-term tracking of plant transpiration	**2020**	[98]
Ionic polymer metal composite	Polymer	**Capacitive**	Pd	**256μF/%RH**	** < 0.5**	22–100%	Nonlinear			**2021**	[146]
Ag/SnO_2_	Metal oxides	**Impedance-type**	Ag/Pd	**6.7 × 10**^**4**^ (Z/Z_0_)	4/6.5	11–98%		2 (11–98%)		**2016**	[79]
Li/K-codoped 3DOM WO_3_	Metal oxides	**Impedance-type**	Ag/Pd	**10**^**5**^ (Z/Z_0_)	15/10	11–95%	Nonlinear	5 (11%-95%)		**2018**	[42]
RGO-BiVO_4_ heterojunction	Nanocomposite	**Impedance-type**		0.98 (Z_0_ − Z_RH_)/Z_0_	3.6/18	11–95%	Nonlinear	**30 (11%-67%)**		**2021**	[80]
Cobalt nanoporous carbon (Co-NPC)/ecoflex	Metal–organic-framework	**Voltage-type**	Ag-covered conductive fabric	0.3 V/%RH		35–80%	**Linear**		Human–robot interactions	**2021**	[35]
TiO_2_ nanowire	Metal oxides	**Voltage-type**	Al		**4.5/2.8**	20–90%	Nonlinear	5 (10%-40%)	Detection of wet targets	**2019**	[87]
rGO	Carbon material	**Voltage-type**	Laser reduced GO	~ 70 mV (V_RH_-V_0_)		25–85%			Touchless devices as artificial skin and flexible panel	**2018**	[169]

Partial work with relatively excellent performance is highlighted in bold

## Performance Enhancement for Flexible Humidity Sensors

To apply the flexible humidity sensors for noncontact measurement, highly sensitive and perdurable devices are desired. However, their performances are still insufficient for practical applications primarily due to relatively low sensitivity, narrow detection range, apparent hysteresis and poor cyclic stability [44, 46]. Meanwhile, compared with other wearable devices, which can be well passivated to record the vital signs and physical activities in real-time, active nanomaterials for the humidity sensors are typically exposed. This may lead to performance degradation after a certain period or even device failure probably due to external scratches, forces or contamination. A thin film-based humidity pass filter laminated on the device is found to well protect the humidity sensor with only slight performance degradation, which is an excellent strategy to passivate the flexible humidity sensor for practical applications [58]. In this section, several viable approaches to improving the humidity sensor performance are overviewed.

### Chemical Doping and Surface Modification

To overcome the low sensitivity of humidity sensors, chemical doping is one of the reliable methods via increasing the hydrophilic sites on active nanomaterials. The common dopants comprise metals [79, 148], ions [42, 149, 150], and nitrogen/oxygen atoms [47, 151, 152]. The dopants are able to effectively tune the electrical property and band structure of sensing materials. For example, the defect can be introduced into GO surface by nitrogen doping with a variety of charge carriers. The hydronium can immobilize the electrons, thus leading to the increase of resistance with humidity increasing [47]. Similarly, another study increases the concentration of H^+^ ions at defect sites to improve the humidity sensitivity via surface doping of nitrogen atoms on mesoporous TiO_2_ structures [48]. The resistance variation of device is over 10^5^ from dry to 90% RH conditions. It is worth noting that the adsorbed oxygen molecules on surface defect sites significantly improve the affinity of sensing materials with water molecules, thereby enhancing the humidity sensitivity.

**Fig. 7:**
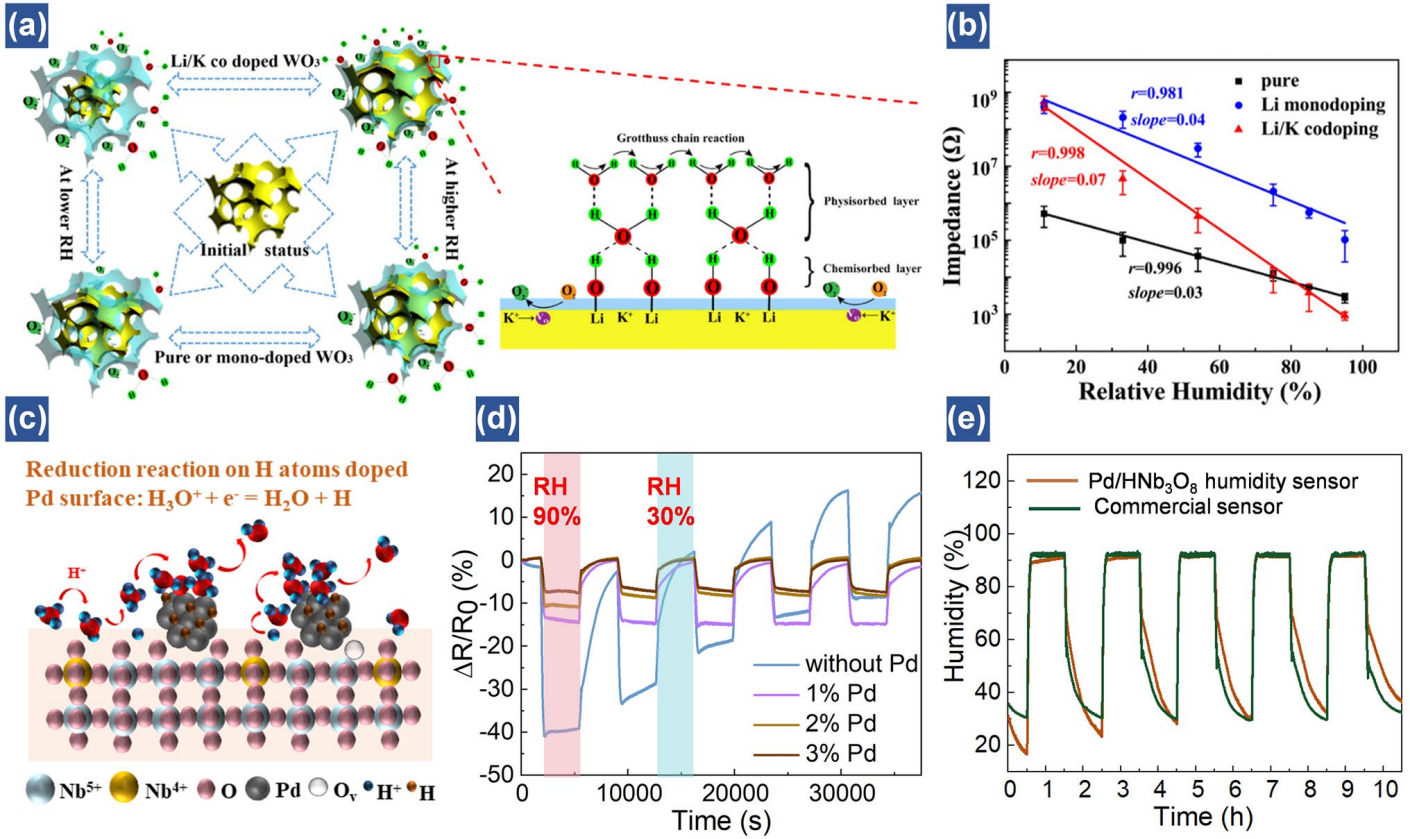
Performance enhancement via chemical doping and surface modification. (**a**) Humidity-sensing mechanism of 3D ordered materials (3DOM) samples. (**b**) Impedance of 3DOM samples under each RH [42]. Copyright 2018 ACS Publications. (**c**) Schematic of the reduction reaction on the H atom assembled Pd/HNb_3_O_8_ nanosheet surface under high RH. (**d**) Cycling tests of HNb_3_O_8_ humidity sensors with different Pd contents and humidity variations from 30 to 90%. (**e**) Long-term cycling tests of a 1% Pd/HNb_3_O_8_ humidity sensor compared with that of a commercial humidity sensor under the same RH [46]. Copyright 2021 Royal Society of Chemistry

To achieve better sensing performance, Wang et al. proposed a Li/K-codoped 3D ordered WO_3_-based humidity sensor with rich structural defects [42]. Owing to the high charge density of Li^+^ ions, water molecules are polarized to promote the generation of H^+^/H_3_O^+^. Meanwhile, the doped K^+^ ions serve as mobile carriers to improve the conductivity. The Li/K-codoped device provides a large number of structural defects and enhances the adsorption of oxygen, which contributes to adsorbing water molecules and then boosting humidity-sensitive performance (Fig. 7a, b). Additionally, the humidity sensitivity of mesoporous-based SnO_2_ after doping Ag nanoparticles is increased by 5.5 times compared to that of only mesoporous SnO_2_ [79]. Therefore, via applying various surface modification methods, humidity sensitivity can be remarkably enhanced primarily due to the increase of charge carriers and narrowed bandgap.

Most of the humidity sensors using dopants usually aim at improving their sensitivity. However, the stability is also of high importance for the practical applications. Lu et al. demonstrated a perdurable flexible Pd/HNb_3_O_8_ humidity sensor by decorating Pd on HNb_3_O_8_ nanosheets [46]. One distinct merit of the composites is that hydrogen atoms on the Pd are able to reduce hydroniums to water molecules and then enhance the reproducibility of sensor (Fig. 7c). As illustrated in Fig. 7d, the pure HNb_3_O_8_ nanosheets-based humidity sensor shows performance degradation with upward fluctuations, although it demonstrates an optimal resistance variation. This is due to that the hydroniums are accumulated on the nanosheets, which hinders the proton hopping process and then weakens reproducibility of the sensor. However, by adding 1% Pd into HNb_3_O_8_ nanosheets, despite of relatively low sensitivity, the stability is quite high due to the generation of high-density hydrogen atoms on Pd surface. A long-time measurement indicates that the output of 1% Pd/HNb_3_O_8_ nanosheets-based humidity sensor almost matches that of the commercially available device (Fig. 7e). On the contrary, Ag and Au nanoparticles-doped HNb_3_O_8_ humidity sensors show pretty poor sensitivity and reproducibility. Actually, the proposed device can work at 90% RH for over 100 h without significant performance degradation. This work provides a guidance to balance the sensitivity and stability of humidity sensors by rationally engineering suitable chemical dopants.

### Structural Design

**Fig. 8:**
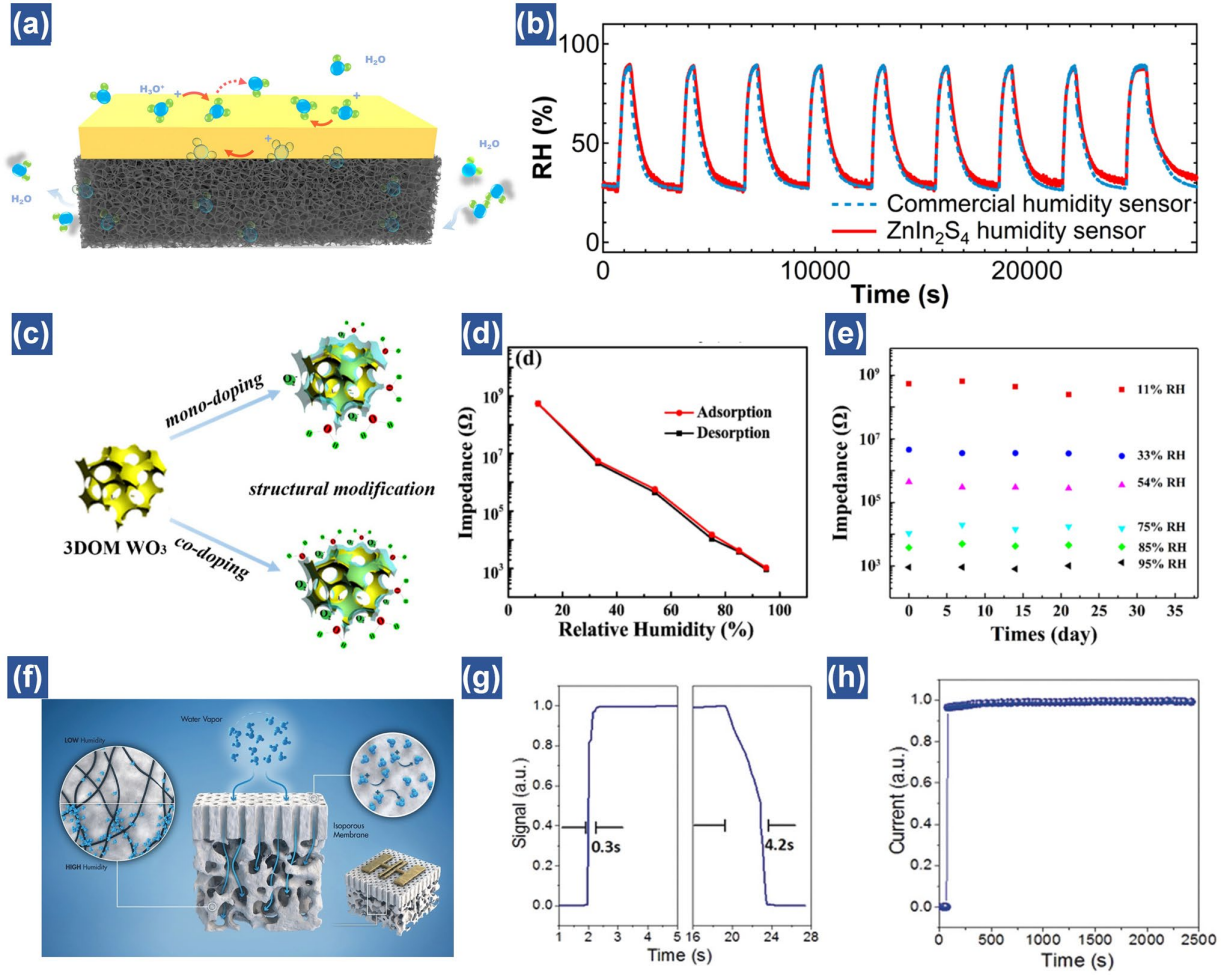
Performance enhancement via structural design. (**a**) ZnIn_2_S_4_ humidity sensor based on LIG porous substrates. (**b**) Long-time cycle measurement results of humidity using a commercial humidity sensor and the proposed humidity sensor in the oven under humidity variations from 30 to 90% [44]. Copyright 2020 ACS Publications. (**c**) Porous structure of Li/K-codoped 3DOM WO_3_. (**d**) The hysteresis plot of the codoped sample. (**e**) Stability of the Li/K-codoped sample [42]. Copyright 2018 ACS Publications. (**f**) Illustration of the sensing mechanism of the CNT-PS-b-P4VP film-based humidity sensor. (**g**) Transient response at 95% RH for carbon nantube-polystyrene-b-poly(4-vinyl pyridine) (CNT-PS-b-P4VP). (**h**) Stability of the CNT-PS-b-P4VP sensor under prolonged humidity (95%) exposure [45]. Copyright 2018 Wiley

With the rapid development of flexible humidity sensors in monitoring human respiration and skin moisture level, it is of great significance to not only achieve high sensitivity, but also fast response and highly steady performance for practical applications. Unfortunately, most of the nanomaterial and polymer-based humidity sensors are generally endowed with relatively thicker intrinsic films and strong hydrophilic nature, inevitably leading to slow response, large hysteresis and poor stability. This is due to the difficulties of water molecules in escaping from the thick and bulk structures [88]. To overcome this obstacle, one of the reliable methods is to rationally engineer active sensing materials or electrodes into porous structures [153, 154]. For example, Lu et al. employed laser induced graphene (LIG) as interdigital electrodes owing to its high-density porous structures, which promote sufficient absorption and desorption of water molecules [44]. Deposition of ZnIn_2_S_4_ nanosheets as active sensing materials onto the LIG electrodes allows the formation of a highly stable humidity sensor. The performance is almost comparable to a commercial counterpart according to the long-time measurement (Fig. 8a, b) [10]. In contrast, using printed carbon and silver electrodes to construct the device, the performance is poor.

For perdurable monitoring, only porous electrodes may not be sufficient for improving the stable performance of humidity sensors based on active relatively thick nanomaterials. This is because of the permeation of water molecules between the layers of sensing nanomaterials, leading to their slow desorption. Recently, tremendous efforts have been devoted to judiciously engineering functional nanomaterials into 3D porous structures utilizing template methods [45], chemical etching methods [155] or hydrothermal methods [156]. Among them, the template method is a desired approach due to its facile and highly productive fabrication processes. For instance, a 3D ordered WO_3_-based humidity sensor was synthesized via a poly (methyl methacrylate) template method, through which a highly uniform pore-structured nanomaterial was successfully obtained (Fig. 8c) [42]. It is worth noted that this WO_3_ humidity sensor presents nearly no hysteresis. Furthermore, even after one month of storage at different humidity levels, this humidity sensor still exhibits highly stable output, indicating superior adsorption and desorption process of water molecules (Fig. 8d, e). Another innovative work demonstrated a new porous composite by embedding CNTs into isoporous block copolymer films. This design realizes a rapid response (0.3 s) and ultrastability of humidity sensor at 95% RH for over half hour (Fig. 8f–h) [45]. Overall, the optimal humidity performance of flexible humidity sensors achieved by elaborate structural designs not only resolves the challenge of water accumulation among layers of thick active nanomaterials, but also prolongs the service life of the devices at wide humidity ranges.

### Joule Heating

Apart from the aforementioned two methods, micro-heaters are supposed as a valuable tool, which can facilitate the physical or chemical desorption process by joule heating to achieve the long-time monitoring, especially at ultrahigh humidities [43, 157–159]. The common metal-based micro-heaters have been applied in not only humidity sensors, but also a variety of gas sensors to further reduce the recovery time. Different from other optimization methods, which mainly focus on the device materials or structures themselves, this strategy is usually realized by integrating a low-voltage electrothermal platform to the humidity device. It was reported that the fully reproducible humidity sensing performance induced by joule-heating is due to the rapid disconnection of physically adsorbed molecules [43]. In most cases, the low repeatability of a humidity sensor often appears at high humidity levels because the water moisture permeates the inner part of the device. As mentioned above, although engineering the structures or nanomaterials into porous architectures provides great merits in improving the response and recovery speed of humidity sensors, it is available only if the humidity sensor is not placed in a high humidity environment for a long time. To address this problem, heating is recommended to rapidly dry the condensed water molecules so as to dramatically reduce the recovery time of humidity sensors. However, heating elements are typically integrated on the rigid material-based substrate (e.g., silicon) due to relatively poor resistance to high temperatures for the flexible/soft materials. This sacrifices the flexibility of devices. Further efforts could be devoted to exploring the high-temperature resistant flexible substrates.

## Flexible Humidity Sensors for Noncontact Monitoring

Flexible humidity sensors, which are sensitive to water molecules, present their unique advantages in noncontact perception. Different from the majority of flexible sensors based on direct contact sensing to track pressure, temperature, strain etc., the noncontact perception can expand their applications in remote sensing or toxic ambient [50, 51, 104]. For example, the COVID-19 pandemic has swept globally since 2020. It has severely disrupted our daily lives. Wearing surgical masks and developing noncontact switches in public especially hospitals have become important means for self-protection and infection prevention [160]. Furthermore, similar to human beings, who rely on skin sweating to regulate the body temperature, the plant is able to manage energy via transpiration processes through stomata on leaves. Such transpiration processes can be accessed in a noncontact way based on the lightweight flexible humidity sensors [55, 57, 161]. Additionally, integrating the humidity sensors with other sensors, flexible printed circuits, smart displays and mobile phones allows the generation of multifunctional portable sensor systems to be endowed with point-of-care feedback functionalities [58, 162–164]. In this section, the applications of humidity sensors in human healthcare monitoring, smart human–machine interactions, plant health status detection as well as feedback system level integration are overviewed.

### Human Healthcare Monitoring

**Fig. 9:**
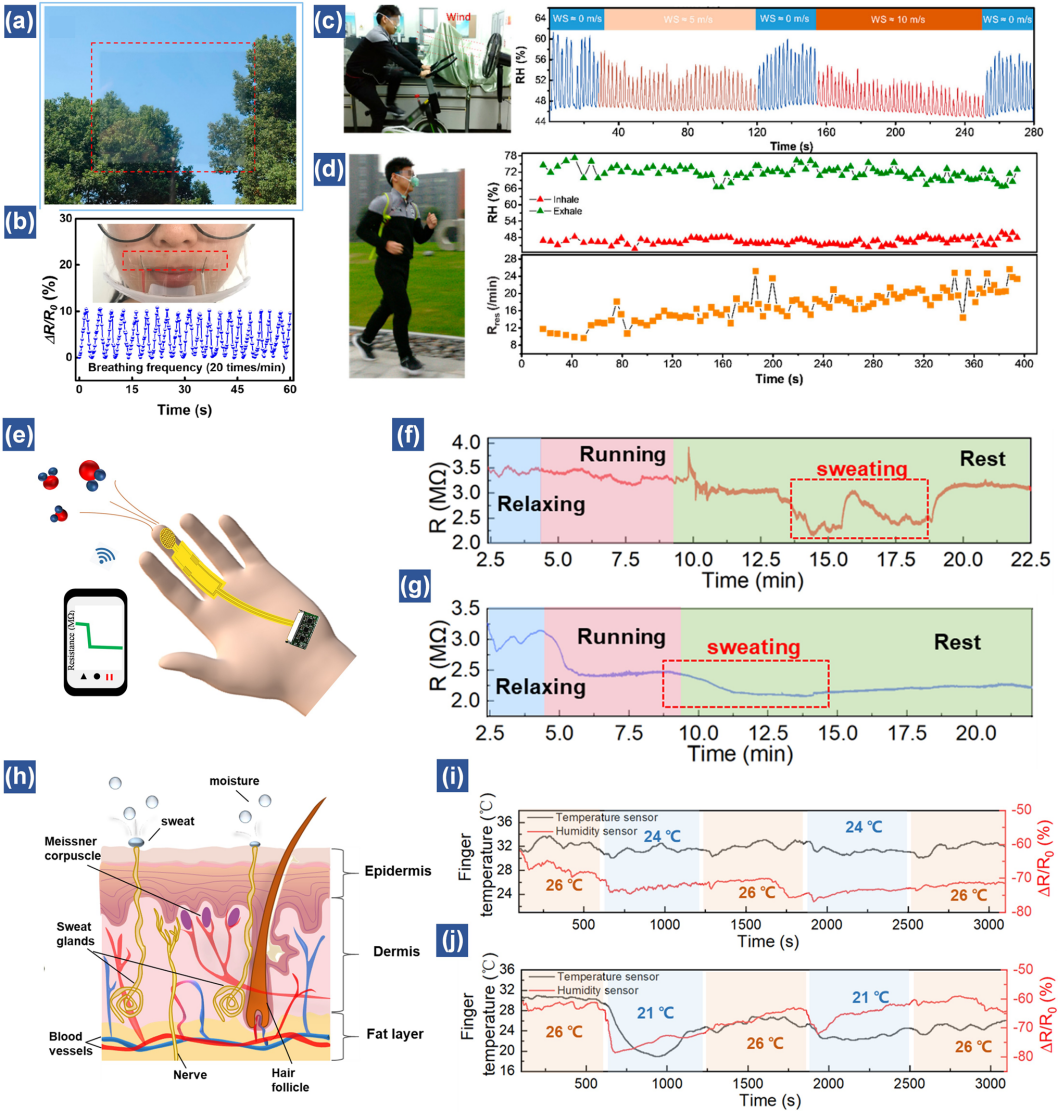
Human healthcare monitoring based on humidity sensors. (**a**) Photograph of cellulose/KOH film. (**b**) Detection of the smooth breathing of an adult, with the inset being the photograph of the respiration test [51]. Copyright 2020 ACS Publications. (**c**) Illustrations of the subject to test the effect of wind speed (WS) to breath signal. (**d**) Real-time respiratory monitoring results of a volunteer during an outdoor jogging exercise (≈3 m s^−1^) [53]. Copyright 2018 ACS Publications. (**e**) Schematic of a wireless finger moisture application. Real-time finger moisture measurement of a subject conducted while running in a (**f**) dehydrated state and (**g**) well-hydrated state [46]. Copyright 2021 Royal Society of Chemistry. (**h**) Schematic picture of a finger-skin structure. Real-time monitoring results of the finger temperature and moisture variations of a volunteer who moved between a warm and a cold room with a temperature difference of (**i**) 2 °C and (**j**) 5 °C [52]. Copyright 2021 Wiley

As the thin film-based humidity sensors are sensitive to moisture variation, various research groups have demonstrated their applications in monitoring human respiration by assembling the flexible sensors on surgical masks [37, 51, 53, 90, 165–167]. For example, in our previous work, by attaching a small piece of HNb_3_O_8_-based humidity sensor on a surgical mask, the respiration rate of a healthy adult was successfully measured, which is about 15 times min^−1^ [46]. To decrease user awareness, a transparent cellulose/KOH film-based humidity sensor was conformally attached onto the curved surface of a plastic face shield to dynamically track the breathing rate (20 times min^−1^) of an adult (Fig. 9a, b) [51]. Furthermore, the humidity sensor can be also applied in indoor and outdoor exercise to evaluate the training strength (Fig. 9c, d). The ambient wind has almost no influence on the breath signals [53].

Besides the respiration monitoring, the humidity sensors can also monitor skin wetness. They can be attached onto different positions of the body to evaluate moisture levels. For instance, Lu et al. demonstrated a perdurable Pd/HNb_3_O_8_ humidity sensor integrated on a glove to record the water loss processes during running (Fig. 9e–g) [46]. The change of finger moisture presents different trends at dehydrated and well-hydrated states. At the dehydrated condition, the finger moisture level exhibits almost no resistance decrease when the volunteer ran for five mins (Fig. 9f). Interestingly, the resistance of humidity sensor decreases obviously after several minutes of rest, indicating that the water is maintained in the body and only released when the body requires cooling. In contrast, the volunteer at the well-hydrated state starts sweating upon running and the resistance further decreases when the subject stopped running due to more perspirations on the fingers (Fig. 9e). The tests suggest that the proposed perdurable humidity sensor can be applied in dehydration diagnosis.

Similarly, by wearing a ZnIn_2_S_4_ nanosheet-based humidity sensor and a CNT/SnO_2_ temperature sensor on the fingertips of five different volunteers, the sudden increase of finger moisture at cold stimulus is observed. This is due to that the sympathetic nerve is stimulated by cold stimulation to secrete sweat. Besides, the thermoregulation center activities of these five volunteers are also monitored. The results reveal that healthy people have the ability of thermoregulation by sweating during exercise. Although this study is still insufficient for diagnosing a targeted disease, it provides a reliable way for physical health management (Fig. 9h–j) [52]. In sum, the wearable humidity sensor affords a potential path for physiological and psychological monitoring based on tracking relative humidity changes on human skin.

### Noncontact Human–machine Interactions

**Fig. 10:**
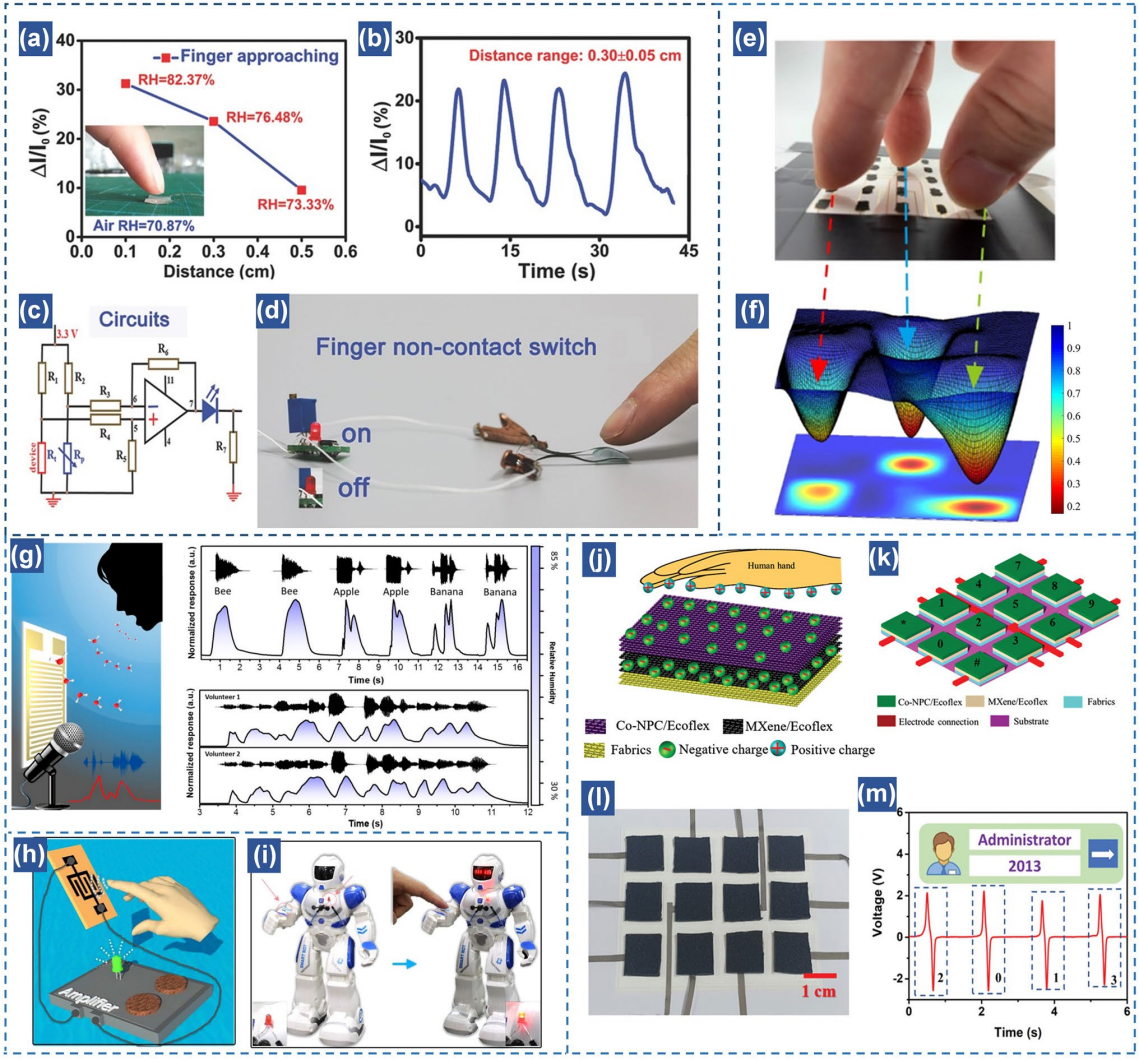
Noncontact human–machine interfaces. (**a**) Measurements of the porous ionic membrane (PIM)-based sensor at different distances between the finger and the PIM (air RH of 70.87%); the inset shows the scene of a finger approaching the PIM. (**b**) The repeatability of the noncontact PIM-based sensor for 0.3 ± 0.05 cm with four circles under air RH of 76.48%. (**c**) The configuration of the noncontact switch circuit system; the sensor resistance was *R*_t_ and the reference resistance was *R*_p_. (**d**) The photograph of a finger noncontact humidity switch system, and it will display the finger approaching by the on–off and degrees of brightness of a LED light [168]. Copyright 2017 Wiley. (**e–f**) 3D mapping of the approaching of three fingertips [75]. Copyright 2019 ACS Publications. (**g**) Schematic illustration of a humidity sensor for human exhaled air detection during speaking [53]. Copyright 2018 ACS Publications. (**h**) Schematic illustration of a visible finger position annunciator. (**i**) IPMEC attached on a robot as artificial skin for the detection of an approaching finger [169]. Copyright 2018 Elsevier. (**j**) Schematic illustration of the Co-NPC/Ecoflex nanocomposite based double-layer non-contact mode TENG. (**k**) Schematic illustration of a smart door lock password authentication system. (**l**) Fabricated prototype of the smart door password authentication system. (**m**) Schematic diagram of the password authentication and output voltage waveform for the CDL-TENG. [35]. Copyright 2021 Wiley

As aforementioned above, our finger surfaces are surrounded by a large quantity of water molecules. Thus, noncontact sensing can be performed when the finger approaches the surface of humidity sensor, which has attracted tremendous research and industry attentions. The RH of humidity sensor presents a gradual increasing tendency if the distance between finger and sensor becomes small. By integrating the device with amplifying circuits, it is able to control the brightness of a LED light based on changing the distance between finger and sensor (Fig. 10a–d) [168]. This indicates the high potentials of flexible humidity sensors in non-contact switches triggered by finger moisture. Another innovative application of flexible humidity sensor is 3D finger moisture mapping, which clearly provides the specific position of approaching fingertips (Fig. 10e–f) [75]. This type of spatial mapping realized by finger moisture distribution promotes the development of moisture-based human–machine interactive systems in a touchless way [100]. Furthermore, integration of transparent humidity sensor arrays on a mobile phone allows to realize contactless and visualized control [50]. The locked or unlocked conditions of mobile phone can be well controlled by a long-range interaction based on identifying the movement trajectory of finger. Another example of humidity sensor for noncontact human–machine interactions is voiceprint recognition anticounterfeiting [53]. Owing to the rapid response of sensor, it is able to accurately record voice tones during speaking or singing near the device by determining the moisture variation. Chen et al. demonstrated the voiceprint recognition of two volunteers by singing the same song, indicating the superior capacity of humidity sensor to recognize human bioinformation features (Fig. 10g) [53]. As it is easy to record, store and imitate human voice by using recording machines, making it unsafe in practical applications, voiceprint recognition realized by humidity sensor is of great significance in practical applications due to that the humidity fluctuation information of different people is quite complex and hard to imitate. Overall, noncontact sensation achieved by flexible humidity sensors enables a highly effective way for safe communication and human–machine interactions in our daily life.

Most of such noncontact applications require continuous power supplies, which may restrict the long-term use in Internet of Things with a large quantity of sensor networks. The chemicals in batteries may also cause environmental issues. Thus, self-powered flexible sensors without external powers are highly appreciated for practical applications. As discussed in Sect. 2, power can be generated by interacting water molecules with active materials and the corresponding output is dependent on the moisture variations around the device. This property enables sensors to realize self-powered function based on monitoring ambient humidity levels [35, 85, 87, 169]. For example, Jiang et al. demonstrated an in-plane moisture-electric converter by assembling GO on rGO electrodes fabricated by laser [169]. An electrical output (~ 70 mV, 12 mA cm^−2^) is achieved when exposing the device in moisture due to ion concentration gradient generated on GO. The self-powered humidity sensor can distinguish the finger position via observing the ON/OFF of LEDs. The sensor can be also attached onto a robot arm as an artificial skin for safe human–robot interactions (Fig. 10h-i). Another important category of self-powered sensors is TENG, which is considered as a green technology based on sequential contact electrification and electrostatic induction [170–173]. In terms of the humidity perception, the output current or voltage of TENG tends to decrease greatly with the humidity increase owing to the absorption of water molecules on the composite surfaces. This leads to the decrease of surface resistance and discharge the triboelectric charges. Therefore, by means of TENG, it is an excellent strategy to develop self-powered flexible humidity sensors [35, 49, 174]. For example, tin disulfide nanoflowers and rGO (SnS_2_/rGO) were hybridized for humidity sensors based on TENG. A steady output voltage up to 24 V is achieved with a wide sensing range from 0 to 97% RH [60]. Furthermore, Park et al. demonstrated a hybrid flexible TENG-based humidity sensor using a metal–organic framework-based cobalt nanoporous carbon coupled with ecoflex as a charge-generating layer and a MXene/Ecoflex as the charge-trapping layer (Fig. 10j–m) [35]. A relative humidity change from 35 to 80% is observed with a linear relationship and the sensitivity is 0.3 V/%. Based on such a self-powered capability, a smart lock password authentication system is proposed. The door can be opened without contacting the door lock using the noncontact interfaces. In short, the self-powered humidity sensor systems open a path towards noncontact human–machine interactions without additional power sources.

### Plant Healthcare Monitoring

With the remarkable improvement of humidity sensing performance, flexible humidity sensors have not only demonstrated their merits in human healthcare monitoring, but also exhibited significance in dynamically tracking plant health status to ameliorate the productivity [44, 175–177]. It is well known that human manage body temperature through sweating [178, 179]. Likewise, plants remove the heat energy via transpiration processes, which rely on stomata on leaves to acquire chemical energy by photosynthesis. Although flexible humidity sensors have been widely employed to monitor human respiration and skin moisture variation, few of them are applied in the plant transpiration monitoring owing to the relatively complex signaling pathways. Tapping into the plant system and accurately capturing the water content require the flexible humidity sensors with high selectivity and perdurable performance.

**Fig. 11:**
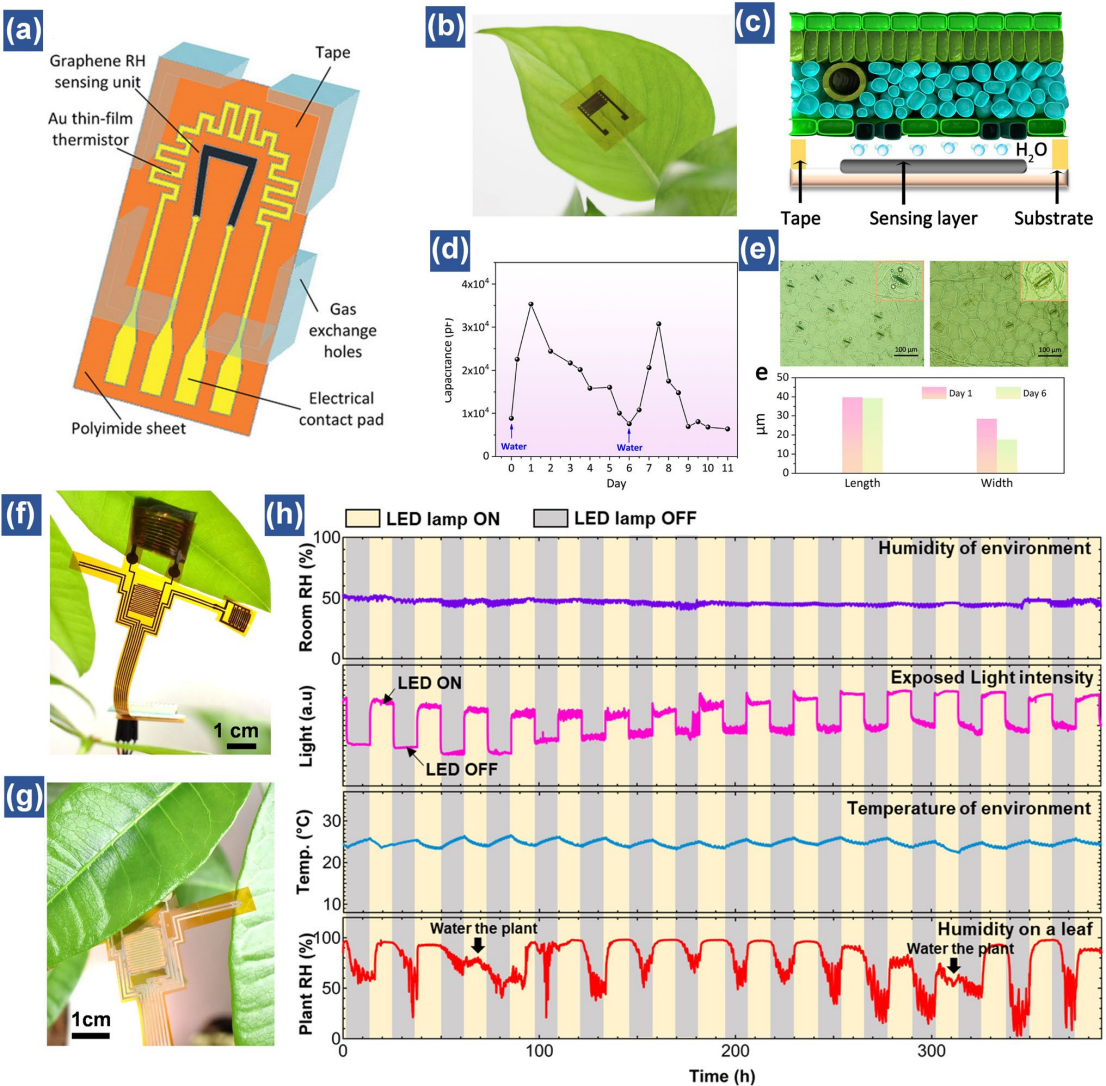
Plant healthcare management. (**a**) Schematic of the integrated temperature and humidity sensor formed on a flexible substrate. The sensor consists of a gold thin-film based temperature sensor and a laser-induced graphene-based RH sensor [161]. Copyright 2021 Wiley. (**b**) Photograph of the GO-based humidity sensor attached to the lower surface of a leaf. (**c**) Schematic illustration of the cross-sectional inner structure of a leaf and the sensing mechanism. (**d**) Real-time monitoring of the capacitance response of the sensor to drought stress over time. (**e**) Images of stomata of the leaf after watering for 1 day (left) and 6 days (right) [98]. Copyright 2020 Elsevier. (**f–h**) Transpiration monitoring results using a multimodal flexible sensor device for over 350 h. Room humidity, light, room temperature, and leaf humidity results are shown from the top to bottom. Monitoring start date: August 08, 2019. Power of artificial light source is 18 W [44]. Copyright 2020 ACS Publications

Generally, tracking the physiological activities of plants is realized by noninvasively epidermal perception. In brief, a flexible humidity sensor sheet is attached onto the lower epidermis with plenty of stomata to dynamically detect the moisture variations throughout day and night. It should be noted that a gap is generally left between the leaf and sensor surface thanks to the advantage of noncontact perception capability of the device. Such a gap also ensures the natural closing and opening of stomata (Fig. 11a). More importantly, considering the large quantity of vapor generated by the leaf during the transpiration monitoring process, this gap can avoid accumulation of water molecules and is advantageous for gas and nutrients exchange [161, 180]. Recently, a flexible humidity sensor fabricated with GO and porous LIG electrodes successfully captured the water status of Epipremnum aureum by attaching the sensor on its lower epidermis of one leaf (Fig. 11b) [98]. It is worth noted that a tape is also used to create a gap between leaf surface and humidity sensor and ensure the normal breath of plant (Fig. 11c). During the transpiration process, the stomata would open and close to release and save the water depending on the temperature of leaves [181–183]. The images of stomata in the 6^th^ day present reduced width of stomata, compared to that in the first day, indicating the drought condition of this plant. However, after resupplying the water, the capacitance of GO-based humidity sensor increases to the original value. This implies that this plant already recovers to the energetic status (Fig. 11d, e) [98]. In addition, plant growth processes are very complex, which is easily affected by various environmental stresses such as light, ambient temperature and humidity, together with the concentration of O_2_, CO_2_ etc. Therefore, an integrated plant sensory system is demanded to measure the growth of plant in a comprehensive way. To obtain detailed information during the plant growth, Hussain et al. designed an integrated device including strain, temperature and humidity sensors. By attaching this integrated sensor sheet onto the leaf surface, the microclimate changes and corresponding physiological signals including elongation, temperature and local humidity of leaves are well recorded [184].

Although the integrated sensor system has been utilized to analyze the growth of plants, the influence of environmental factors such as light and temperature is insufficiently investigated. During photosynthesis and transpiration process, light is one of the key factors that affects nutrients and water exchange with outside environment. Once the plant is exposed under sunlight, the surface temperature of leaves would increase. In this case, the transpiration process of plant is triggered to maintain a suitable leaf temperature. To confirm this, a multimodal plant healthcare system consisting of two ZnIn_2_S_4_ humidity sensors, a CNT/SnO_2_ temperature sensor and a ZnIn_2_S_4_ optical sensor is proposed. Among them, one of the humidity sensors is used to detect humidity level of plants and the other is for measuring the environmental humidity. The ZIS nanosheets-based optical sensor is exposed to the simulated sunlight, which is able to simulate day and night by automatically switching on and off every 12 h. Owing to lightweight (0.3 g) and excellent flexibility of this device, it could be conformally attached onto the leaf surface (Fig. 11f–g) [44]. The transpiration processes of *P. microcarpa* and *Ficus macrocarpa* are successfully monitored using this proposed sensor system. Notably, during the measurement, two drought conditions of *P. microcarpa* are observed. However, the humidity level of detected leaf recovers to an original value after watering. Moreover, the whole monitoring process takes around 16 days, during which all the sensors work normally without significant performance degradation (Fig. 11h). In short, the outstanding sensing performance of flexible humidity sensor-based system enables the pre-diagnosis of plant dehydration, which opens pathways toward improving crop/plant productivity. In combination with technologies of internet-of-things and big data, it is possible to realize smart agriculture in near future.

### Integrated Humidity Sensor-based Feedback Systems

**Fig. 12:**
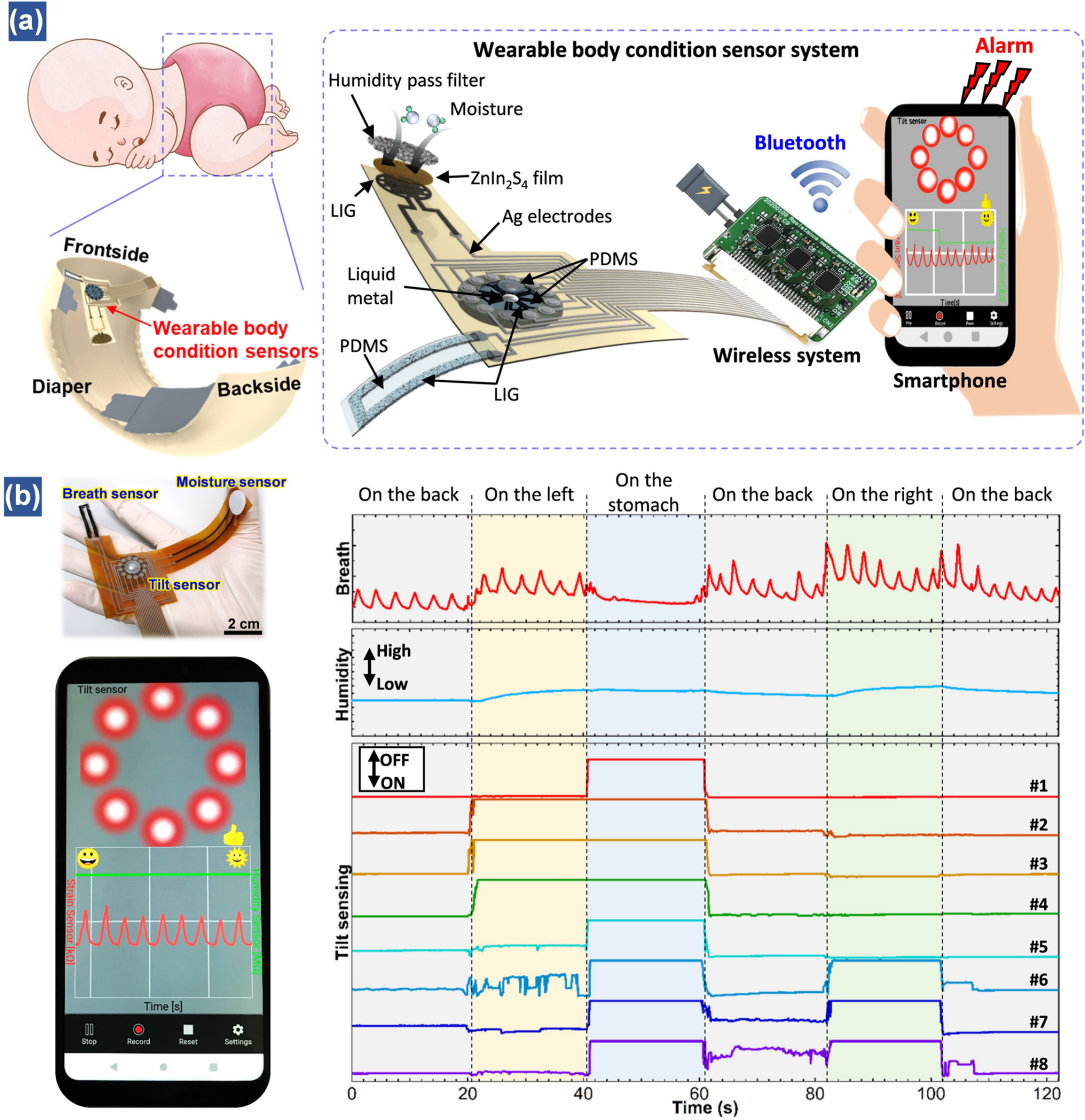
Multimodal humidity sensor-based systems with feedback functions. (**a**) Schematic of the wearable sensor system attached onto a disposable diaper, the sensor system is integrated with tilt, breath, and moisture sensors. Multiple channel signals are wirelessly transmitted to a smartphone interface via Bluetooth. The smartphone can generate an alarm under certain conditions. (**b**) Wireless real-time monitoring results measured from an adult lying on a mattress [58]. Copyright 2021 Wiley

Recent advances in wearable electronics tend to develop multifunctional integrated flexible sensor systems, which not only comprise multimodal sensors or sensors array to provide various functionalities, but also involve signal transduction, processing and wireless transmission to a smartphone interface for point-of-care detections with instant feedback information to users [185–188]. Such point-of-care measurements are especially helpful to vulnerable groups such as the elderly and neonates who usually need special cares. For instance, the baby diaper wetness often depends the sleeping quality of infants. Thus, how to smartly track the diaper moisture is of importance in improving the baby’s sleeping comfortability. Xu et al. applied the humidity sensor onto the baby diaper to evaluate the diaper moisture in real time [58]. If the urine approaches the device and the output of humidity sensor reaches the threshold, alarm music can be induced to remind the guardians to change the diaper for infants. Furthermore, the humidity sensor is integrated with tilt and strain sensors to monitor the diaper wetness, sleeping positions and breathing rate of infants, respectively (Fig. 12a). The multimodal sensor system can be coupled with a micro-controller and smartphone, which is programmed with visualized interfaces and alarm feedback functions. If the neonate sleeps in a prone posture which may cause sudden death due to a lack of oxygen or respiratory infection, the smartphone can generate alarm music. Besides, the abnormal breathing rate of babies and high humidity of diapers are also able to trigger alerts to inform the caregivers (Fig. 12b). In short, such feedback hybrid sensor systems based on the humidity sensor, various functional multimodal sensors and portable circuits as well as smartphones enable to realize point-of-care feedback detections in our daily lives.

## Conclusions and Outlook

The past decade has witnessed tremendous progress in a variety of skin-like flexible sensors to track physical, chemical and biochemical signals. As an intriguing type of flexible sensor with the distinct merit of noncontact monitoring, flexible humidity sensors exhibit their diverse applications in human healthcare, plant transpiration and human–machine interactions. Owing to the high-performance flexible humidity sensors, noncontact human respiration and human–machine interfaces contribute to reducing the risk of cross-infection among different users, especially during the COVID-19 epidemic. In this review, first, we have critically discussed the recent innovative flexible humidity sensors based on various functional materials. To meet the requirement of perdurable applications, various optimization strategies mainly based on surface modification, structural design and Joule heating are then overviewed to improve the sensitivity, detection range, response/recover speed and stability of humidity sensors. Based on the high-performance flexible humidity sensors, several typical examples of humidity sensors in contactless detections are introduced from unitary functionality to integrated hybrid feedback sensor systems. These multifunctional wearable humidity sensor systems afford a new path toward noncontact perceptions, which expand their applications in remote sensing or toxic ambient.

Although enormous achievements have been made in flexible humidity sensors, a couple of crucial factors should be considered to fulfill the practical applications. For instance, most of the current proposed flexible humidity sensors are hard to operate for a long time, especially at high humidity levels (> 90%) due to tough desorption of water molecules from the device. This drawback limits their perdurable applications, although porous structures or Joule heating can sustain the performance to some extent. An effective strategy relies on the strong reducibility of hydrogen atoms on the Pd surface to regulate hydroniums, which achieves a highly stable humidity sensor for over 100 h (90% RH) almost without performance degradation [46]. Further research may focus on exploring new sensing mechanisms and discovering innovative nanomaterials to address the unstable issues of thin film-based humidity sensors. Second, a variety of research groups have been involved in elevating the intensity of power output driven by moistures. More efforts are encouraged to develop self-powered humidity sensors for wearable green electronics [85]. Third, most of the flexible humidity sensors rely on solution-based methods, which are relatively hard to achieve reproducible and stable devices from batch to batch. Lithography patternings or laser direct writing approaches afford more reliable properties of humidity sensors. In particular, by tunning fabrication modes, laser processing is able to simultaneously create electrodes as well as active materials in response to moistures. Such facile, highly repeatable and high-throughput means push the flexible humidity sensors for commercialization. Last but not the least, from the perspective of multimodal integrated sensor systems, signal cross-coupling effect should be considered. As humidity may influence the property of many functional materials, the humidity effect on other sensors integrated on thin films should be well investigated. Future efforts from intimate cooperation of interdisciplinary researchers should be able to bridge the gap between the high-performance flexible humidity sensor system and practical applications toward perdurable noncontact measurements.
